# On the road to sustainability: Applying an extended Theory of Planned Behaviour model to energy-saving transportation practices

**DOI:** 10.1371/journal.pone.0325196

**Published:** 2025-06-03

**Authors:** Anda-Bianca Ciocîrlan, Richard Rowe

**Affiliations:** School of Psychology, University of Sheffield, Sheffield, United Kingdom; East China Normal University, CHINA

## Abstract

Mitigating climate change demands urgent action, particularly in reducing CO2 emissions, a major contributor to global warming. Individual behavioural changes in transportation patterns are needed to lower environmental impact. To design interventions that can target these behaviours, it is essential to understand the beliefs that underlie them. This study uses an extended Theory of Planned Behaviour model to identify the beliefs underlying sustainable transportation practices. Five behaviours were included: (i) general sustainable transportation, (ii) public transportation, (iii) walking and cycling, (iv) reducing car use, and (v) reducing flights. A three step-approach was employed. First, beliefs underlying the Theory of Planned Behaviour variables were elicited using qualitative methodology. The most commonly stated beliefs were included in the second phase; a questionnaire study that measured beliefs, attitudes, subjective norm, perceived behavioural control, habit, moral norms, and intention. Lastly, a one-week follow-up measured behaviour. Behavioural beliefs strongly predicted attitudes, habit beliefs predicted habit, and moral norm beliefs predict moral norm across all five behaviours. Regression models showed that the extended Theory of Planned Behaviour model had a better predictive capacity than the standard model. The strongest predictors were habit and moral norm. Intention significantly predicted behaviour but only explained a low proportion of variance. Interventions aiming to promote sustainable transportation practices should particularly focus on influencing individuals’ habits and moral norms, as these factors impacted most behaviours studied.

## Introduction

### Background

The transportation sector is one of the main contributors to global CO2 emissions [1], and the largest emitting sector in the United Kingdom [2]. Twenty-four percent of the total greenhouse gas emissions recorded in the United Kingdom can be attributed to transportation. The least sustainable transportation modes are planes, motorbikes, petrol and diesel cars. Trains, electric cars, and coaches are more sustainable alternatives for intercity travel, as they emit up to three times less CO2 for a single passenger [2].

While the Paris Agreement [3] has set the target of achieving the Net Zero Emissions goal by 2050, its feasibility remains uncertain under the current circumstances. The International Energy Agency (IEA) [4] emphasised that even though the number of countries that pledged to achieve the goal has increased over the last years, most pledges are still awaiting the implementation of policies and measures to support them. The IEA highlights the importance of behavioural changes for achieving the Net Zero Emissions goal [5]. Behavioural changes in transportation patterns that are necessary to make significant progress include increasing public walking, cycling, and taking public transport instead of driving. Psychological science plays a key role in understanding the mechanisms underlying behaviour change and can help inform interventions promoting sustainability.

Several psychological theories have been developed to model factors that influence engagement in specific behaviours. The Value-Belief-Norm (VBN) theory [6] proposes that individuals are more likely to engage in pro-environmental behaviours when they hold strong ecological values, believe that environmental issues pose a threat to things they care about, and feel a moral obligation to act. This highlights the role of personal norms and sense of responsibility. Similarly, the Theory of Reasoned Action (TRA) explains behaviour as influenced by intentions, which are determined by attitudes and subjective norms [7]. However, a limitation of the TRA is that it assumes behaviours are under volitional control and does not account for any external barriers. To address this limitation, Ajzen [8] extended the TRA by developing the Theory of Planned Behaviour (TPB), which introduced perceived behavioural control.

The TPB is a commonly applied psychological framework aiming to explain behaviour. It has been effective in explaining a wide-range of behaviours, such as alcohol and tobacco use [9] and physical activity [10]. The TPB posits that attitudes towards a behaviour (i.e., the degree to which performance of the behaviour is positively or negatively valued), subjective norms (i.e., the perceived social pressure to engage or not to engage in a behaviour), and perceived behavioural control (i.e., people’s perceptions of their ability to perform a given behaviour) are determined by three types of beliefs. Behavioural beliefs are beliefs about outcomes or experiences produced by a behaviour, and they influence attitudes. Normative beliefs are beliefs about other individuals’ behaviour or approval of the behaviour, and they determine subjective norms. Control beliefs are beliefs about the presence or absence of factors that can facilitate or impede a behaviour, and they influence perceived behavioural control. Attitudes, subjective norms and perceived behavioural control collectively influence intention to perform behaviour, and ultimately, intention is modelled to be the most proximal predictor of behaviour. Research applying the TPB [11] to sustainability behaviours has grown over the years. Recent research using the TPB supports its application to sustainable transportation [12–15]. For instance, a study [12] investigated the intention to adopt electric vehicles in Jordan using the TPB. They found that attitude, subjective norm and perceived behavioural control have a significant and positive effect on intention, and the TPB model had a good level of explanatory power, accounting for 62.4% of the variation in intention. Similarly, another study [13] found that attitude, subjective norms, and perceived behavioural control had a significant impact on citizens’ intentions to use public transportation, with attitudes playing the most important role. Yeğin and Ikram [14] found that an extended TPB model, including environmental concerns and green trust, was effective in explaining consumers’ intentions to purchase electric vehicles.

Extensions to the TPB have been developed that aim to increase predictive power. A common additional construct is habit. Habit is defined as systematic repetitions of a behaviour that lead to reinforced consequences [16]. Habit has been found to be a significant predictor of transportation mode choice. De Brujin et al. [17] found habit strength was the strongest independent predictor of bicycle use after controlling for TPB variables. Interaction analyses showed that intention significantly predicted bicycle use for people with low habit strength, but not for people with high habit strength. Other studies also supported the inclusion of habit in TPB models. For example, Donald et al. [18] and Verplanken et al. [19] found that habit was a significant predictor of car use.

Moral norms can also be added to the TPB model to explain sustainable behaviours. Moral norms represent individuals’ responsibility to engage in a specific behaviour [20]. Unlike subjective norms, moral norms are not influenced by external factors, but represent a sense of responsibility towards the environment in the context of pro-environmental behaviour. It has been found that moral norms were the most influential determinant of waste separation behaviour [21]. In sustainable transportation, moral norms have also been found to improve prediction of behaviour beyond the classic TPB predictors. For instance, moral norms were found to explain variance in bus use independently from the other TPB [22]. Shalender and Sharma [23] investigated the role of moral norms in predicting intention to adopt electric vehicles in India and found that moral norms, alongside the other TPB constructs, significantly predicted consumers’ intentions. The findings suggest that feeling a strong moral obligation to reduce environmental impact may lead to increased engagement in pro-environmental behaviours.

One limitation of previous research is that numerous studies use the TPB to explain intention, but do not extend the investigations to predict behaviour [11]. This practice leaves an important gap in understanding how intentions ultimately translate into behaviour. The Intention-Behaviour Gap refers to the discrepancy between what individuals intend to do and what they actually do [24]. While the TPB suggests that intentions are the main determinant of behaviour, it has been consistently shown that intentions do not always translate into actions. A meta-analysis of experimental evidence investigating how changes in intentions determine changes in behaviour at a later time point [25] found that a medium-to-large change in intention (d = 0.66) leads to a small-to-medium change in behavior (d = 0.36). This gap may be influenced by habit, personality, socio-demographic factors, and external constraints [26]. As a result, TPB studies that measure intentions and not behaviour may not provide a reliable basis for intervention development, as they do not capture whether intentions translate into action. Addressing this limitation requires longitudinal studies that include a measure of subsequent behaviour.

Another limitation is that the belief elicitation stage is often omitted in TPB studies [27]. Application of the TPB can involve measuring the specific beliefs that underlie the TPB concepts. Identifying these beliefs can inform intervention design of the key beliefs to target to change intentions and behaviour. Interventions can be tailored to strengthen positive beliefs and challenge negative beliefs in order to change intentions and behaviours. For instance, if individuals believe that sustainable food consumption is expensive (a behavioural belief), then interventions can be designed to challenge this belief, with the aim of making attitudes towards the behaviour more positive, and therefore strengthening intentions to eat more sustainably.

### Current research

The current study applies the TPB to sustainable transportation behaviours, addressing the limitations discussed above. First, while many studies have used the TPB to predict intentions, fewer have extended this to actual behaviour. This research investigates behaviour with a follow-up. Second, the study incorporates the belief elicitation stage, which is often omitted in TPB research. The belief elicitation stage promotes the understanding of the specific beliefs that influence intentions, subjective norms, and perceived behavioural control. Third, additional variables are added to enable the comparison of the standard TPB model and an extended TPB model.

**Fig 1:**
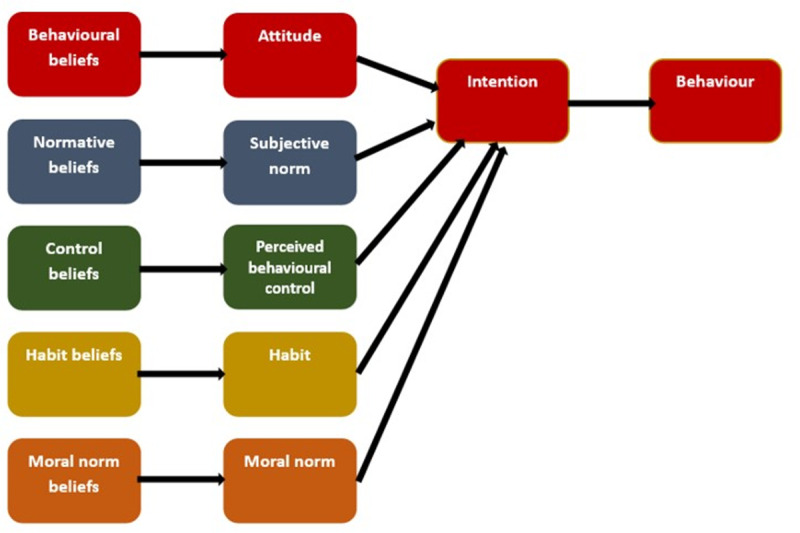
Expected model based on hypotheses.

The current study aims to investigate the factors underlying five behaviours related to sustainable transportation: (i) general sustainable transportation practices, (ii) public transportation, (iii) walking and cycling, (iv) reducing car use, (v) reducing flights. Developing an understanding of these factors will support the development of interventions that target the specific constructs that have the highest influence on intention and behaviour. An extended TPB model is used, adding habit and moral norm as additional predictors (see Fig 1).

For each of the five behaviours, the following hypotheses will be tested: (1) Beliefs are associated with the extended TPB construct that they determine (i.e., behavioural beliefs are associated with attitudes, normative beliefs are associated with subjective norms, control beliefs are associated with perceived behaviour control, habit beliefs are associated with habit, and moral norm beliefs are associated with moral norms), (2) the extended TPB variables (i.e., attitude, subjective norm, perceived behavioural control, habit, moral norm) are associated with intention to adopt behaviour, (3) intention is prospectively associated with behaviour, (4) the extended TPB model explains a greater proportion of the variance in intention than the classic TPB model.

Hypothesis (3) will not be tested for flight reduction as it would not be practical to measure this behaviour with a one-week follow-up.

## Method

### Design and procedure

The present study was pre-registered on the Open Science Framework (https://osf.io/5bh9k) and received approval from the research ethics committee of the School of Psychology, University of Sheffield (056001). The study was conducted online, using Qualtrics [28], a survey platform that allows secure remote data collection. Participants were recruited from Prolific [29]. Participation was voluntary and the participants were informed prior to accessing the questionnaires that the study investigated sustainable transportation. They were paid in line with the Prolific requirements. When they signed up for the study, they were shown an Information Sheet that provided details of the study, what would be required of them, confidentiality, how the data would be used, and further information for queries or complaints (see S1 File).

The study used a mixed-methods design combining both qualitative and quantitative elements to investigate five behaviours related to sustainable transportation (i.e., general sustainable transportation, reducing car use, reducing flights, walking and cycling, and public transport use). Different samples were collected for each one of the behaviours. The study was divided into three parts:

1. In the qualitative elicitation phase participants were asked open-ended questions to elicit beliefs about the target behaviour to inform the development of belief items in the main questionnaire. Content analysis involving two independent coders identified the range and frequency of the beliefs elicited.
2. A quantitative questionnaire measured demographic variables, TPB variables (i.e., beliefs, attitude, subjective norm, perceived behavioural control, and intention), and extended TPB variables (i.e., moral norm, and habit) for each of the five target behaviours.
3. A follow-up was conducted after one week. The participants who completed the main questionnaire were invited to take part. The follow-up consisted of one question measuring participants’ self-reported behaviour in the last week.

The elicitation phase took 5 minutes on average, the main questionnaire took 6 minutes on average, and the follow-up took 30 seconds. After completing the questionnaires (i.e., both main questionnaire and follow-up), the participants were provided a link to the Energy Saving Trust website [30] for further information about sustainability and energy saving.

### Participants

The participants recruited in all research phases were residents of the United Kingdom. Participants were recruited via Prolific an online recruitment platform used in psychological research. This platform was selected as it provides high-quality data and access to diverse, pre-screened participant pools. It has been demonstrated that Prolific samples are more likely to pass attention checks and provide higher data quality (e.g., more meaningful answers, follow instructions, read all the items) than alternative recruitment platforms [31]. Relevant in-built filters were used to ensure that the participants matched the inclusion criteria: for the car reduction group, only car drivers were allowed to take part, while for the flight reduction group, only regular flyers were invited. Twenty-five participants were recruited to take part in the elicitation study for each behaviour; 125 participants were recruited in total for the elicitation phase. The number of participants recruited was motivated by the objective of achieving data saturation, a point where further data collection is unlikely to substantively alter the findings, as suggested by guidelines for elicitation studies [32]. The participants were 64% female and 36% male, with a mean age of 37.65 years (SD = 12.23, range = 18–71).

For the main questionnaire phase, an a priori power analysis was conducted using G*power 3.1.9.7 [33] for F tests, specifically linear multiple regression with a fixed model and R^2^ deviation from zero. The results indicated that 98 participants were required for each behaviour to detect an effect size of d = 0.30, with alpha set at.05 at 95% power. The selected effect size of 0.30 was based on a meta-analysis investigating the efficacy of the TPB [34] which included 185 studies addressing a wide-range of behaviours, including physical activity, substance use, and eating behaviours.

A total of 490 participants were recruited, with 98 participants being recruited in each group. The participants were 54.3% female, 45.3% male, and 0.4% other, with a mean age of 39.94 years (SD = 13.46, range = 18–80).

To measure behaviour, the participants who took part in four of the behaviour groups (i.e., sustainable transportation, public transportation, walking and cycling, and car reduction) were invited to take part in a follow-up study one week after completing the main questionnaire. Of the 392 participants invited to take part in the follow-up, 346 (88%) completed it. The participants’ age and gender are displayed in Table 1.

**Table 1:** Demographic information.

Research phase	Gender			Age			
	M (%)	F (%)	Other(%)	Mean	SD	Min	Max
**Belief elicitation**	45 (36%)	80 (64%)	–	37.65	12.23	18	71
**TPB questionnaire**	222 (45.3%)	266 (54.3%)	2 (0.4%)	39.94	13.46	18	80

### Measures

The present study follows guidance on constructing questionnaires based on the Theory of Planned Behaviour [32,35]. However, modifications were made to include the features of the extended TPB model and the specific research context as detailed below.

### Belief elicitation phase

A questionnaire was developed for each one of the five behaviours investigated. The questionnaires consisted of 14 open-ended questions, eliciting behavioural beliefs, normative beliefs, control beliefs, habit beliefs, and moral norm beliefs. The commonly identified beliefs elicited in this phase were used to construct the TPB questionnaires. Recommendations from Ajzen and Fishbein [7] were followed in identifying the final set of beliefs to be included in the main questionnaire, the beliefs having to appear in 10% of the answers. Two coders independently coded the beliefs, and the agreement percentage for each belief ranged from 86% to 98%. Disagreements were resolved through discussion.

### Theory of Planned Behaviour questionnaire

A TPB questionnaire was developed for each one of the target behaviours, measuring beliefs (as identified in the elicitation phase), TPB constructs (i.e., attitudes, subjective norms, and perceived behavioural control), and extended TPB variables (i.e., habit and moral norm). Demographic data was also collected, including sex, age, ethnicity, and education.

The behavioural, normative, control, habit, and moral norm beliefs that were identified in the elicitation phase were measured on 7-point Likert scales. Participants rated their agreement with the statements from strongly disagree to strongly agree.

The TPB behaviour constructs were measured as recommended by Ajzen [35]. Responses to each item ranged from 1 to 7. Higher values indicated stronger positive attitudes, stronger endorsement of subjective norms, stronger behavioural control, higher intention, stronger habit, and stronger endorsement of moral norms.

Attitudes were measured with seven-point semantic differential scales in terms of the following dimensions: (i) unpleasant – pleasant, (ii) unhealthy – healthy, (iii) detrimental – beneficial (e.g., Choosing public transportation on a regular basis would be…), and two seven-point Likert scales asking participants to rate their agreement with the target behaviour being valuable to them, and being a wise decision. Alpha reliabilities ranged from 0.83 to 0.90 across the five behaviours.

Subjective norms were measured on a seven-point Likert scale where participants rated their agreement, from strongly disagree to strongly agree, with four statements. An example of a statement is: “Most people who are important to me approve of me choosing sustainable transportation practices on a regular basis”. Alpha reliabilities ranged from 0.71 to 0.77 across the five target behaviours.

Perceived behavioural control was measured using the mean of four items, rating agreement on a seven-point Likert scale. For instance, the participants were shown the statement: “I believe I am capable of choosing sustainable transportation practices”, and asked to select their agreement from strongly disagree to strongly agree. Alpha reliabilities ranged from 0.70 to 0.90 across the five target behaviours.

Habit was measured using the mean of two items rating agreement on a seven-point Likert scale, from strongly disagree to strongly agree. For instance, regarding sustainable transportation, the participants rated the following statements: (i) “Choosing sustainable transportation practices has become my natural choice”, and (ii) “I frequently choose sustainable transportation practices”. Alpha reliabilities ranged from 0.85 to 0.93 across the five behaviours.

Moral norms were measured using the mean of two items rating agreement on a seven-point Likert scale, from strongly disagree to strongly agree. For instance, in the questionnaire addressing sustainable transportation, the participants rated the following statements: (i) “It would feel right for me to opt for general sustainable transportation practices”, and (ii) “I would feel guilty if I did not opt for general sustainable transportation practices”. Alpha reliabilities ranged from 0.62 to 0.85 across the five target behaviours.

Intention to perform the target behaviour was measured using the mean of four items, rating agreement on a seven-point Likert scale. For instance, the following statement was presented: “I would like to choose sustainable transportation practices on a regular basis”, and agreement was measured from strongly disagree to strongly agree. Alpha reliabilities ranged from 0.93 to 0.94 across the five target behaviours.

### Follow-up

Ultimately, behaviour was measured at one week follow-up. For instance, participants in the car reduction group were asked “How much have you walked and/or cycled in the last week?”. The responses were categorised in (i) never, (ii) occasionally, (iii) often, (iv) very often, and (v) always.

See S2 File for the complete questionnaires used in the present study.

### Data analysis

R version 4.2.1 [36] running on a Windows 10 PC was used for data preparation and statistical analysis. The beliefs elicited in the belief elicitation phase were identified using content analysis by two independent coders. Exploratory factor analyses were conducted separately for behavioural, normative, control, moral norms, and habit beliefs to combine related beliefs into factors. A Promax rotation was employed to allow the emergent factors to be correlated. The number of factors to be extracted was determined by inspection of eigenvalues, scree plots, and parallel analysis scree plots, which compare the eigenvalues from the actual data to the eigenvalues obtained from random data [37]. Factor interpretability was used as an additional criteria when the number of factors suggested by each method was different. Belief scales were formed by adding up the scores of items with loadings higher than.3, and the reliability of the scales was examined with Cronbach’s alpha. Items loading highly on more than one factor (i.e., cross-loadings) were omitted.

Cohen’s [38] conventions were employed for interpreting the strength of correlation coefficients, coefficients being classified as small (.20), medium (.50), or large (.80). The data collected in the main questionnaire and in the follow-up were analysed using regression models. Models were fitted to analyse the following: (i) the extent to which behavioural beliefs, normative beliefs, control beliefs, habit beliefs, and moral beliefs predicted attitude, subjective norm, perceived behavioural control, habit and moral norm, (ii) the extent to which attitude, subjective norm, and perceived behavioural control predicted intention, (iii) the extent to which the extended TPB model, including habit and moral norm predicted intention, and (iv) the extent to which intention predicted target behaviour.

## Results

### Exploratory factor analyses of belief items

Correlations between belief items ranged from small to large within the sets of behavioural, normative, control, moral, and habit beliefs for the five behaviours investigated (see S3 File). Given the mixed correlations observed, additional tests were conducted to assess the suitability of the data for exploratory factor analyses. Bartlett’s test of sphericity was significant, indicating that the variances are not all equal and correlations exist. Additionally, the Kaiser-Meyer-Olkin (KMO) measure was used to assess the proportion of variance in the belief items that may be caused by underlying factors. The KMO statistics showed that the variables had high contributions, suggesting the data is appropriate for factor analysis, with exceptions discussed below. These tests showed that the data regarding all five behaviours were suitable for exploratory factor analyses. The results of the factor analyses are displayed in S4 File.

### Sustainable transportation

A two factor solution was chosen for behavioural beliefs, consisting of advantages (e.g., help the environment) and disadvantages (e.g., make me worried). The KMO measure showed that four of the normative beliefs did not have a high enough contribution, with values less than.5, so they were discarded. A three factor solution was chosen for normative beliefs, consisting of disapprovers (e.g., the motor industry), young approvers (e.g., students) and professional approvers (e.g., businessmen). The alpha reliabilities increased for two of the factors by removing two items. Control beliefs were split into two factors: barriers (e.g., inconvenience) and facilitators (e.g., increased efficiency). A unifactorial solution was found to be appropriate for both the habit (e.g., I tried sustainable transportation and it was a pleasant experience) and the moral norms beliefs (e.g., values influence decision to choose sustainable transportation). The alpha reliabilities across the beliefs were mostly acceptable to good, with a few excellent values, ranging from 0.77 to 0.92.

### Public transportation

A four factor solution was chosen for the behavioural beliefs. Seven items loaded highly onto a disadvantages factor, such as public transport being unreliable and inconvenient. Three items loaded strongly onto an environmental benefits factor (i.e., help the environment, reduce carbon footprint, less air pollution). Three items loaded highly onto a positive experience factor, including enjoyment and relaxation. Factor 4 was made up of 2 items representing practical advantages (i.e., convenience, lower transportation costs). The analysis of normative beliefs identified three factors: approvers (e.g., commuters), positive role models (e.g., environmentalists), negative role models (e.g., drivers). The control beliefs analysis identified facilitators (e.g., frequency), ineffectiveness (e.g., low accessibility), and inconvenience (e.g., time consuming). A two factor model provided a good fit for habit beliefs, consisting of experience (e.g., I tried public transportation but it was inconvenient) and automatic choice (e.g., I find myself automatically choosing public transportation when I go on a day or weekend trip). A one factor solution was found for moral norm beliefs, representing moral responsibility, as only one eigenvalue was above 1 and inspection of the scree plot also pointed to a single factor. The alpha reliabilities across the beliefs ranged from 0.70 to 0.89, values indicating acceptable to good reliability.

### Walking and cycling

A two-factor solution was chosen for the behavioural beliefs, consisting of advantages (e.g., improve health) and disadvantages (e.g., it is time consuming). Normative beliefs were divided into four factors: positive role models (e.g., fit people), approvers (e.g., young people), disapprovers (e.g., elderly) and negative role models (e.g., people with limited mobility). Three-factor solutions were picked for both control and habit beliefs. Control beliefs were split into facilitators (e.g., better infrastructure), personal barriers (e.g., health issues) and external barriers (e.g., bad weather). Habit beliefs were represented by automatic choice (e.g., I found myself automatically choosing sustainable transportation when I go shopping), positive experience (e.g., I tried walking and/or cycling and enjoyed it), and negative experience (e.g., I tried walking and/or cycling but I could not cope due to my health). Only two items loaded highly onto positive experience and only two items loaded highly onto negative experience. However, a three-factor model was found most suitable as three of the eigenvalues were above 1, and the one-factor solution suggested by the scree plot would have required the removal of the two negative experience items. A unifactorial solution was chosen for moral norm beliefs, representing responsibility (e.g., I want to walk and/or cycle because I value my health and body). Most of the alpha reliabilities were acceptable, good, and excellent from 0.74 to 0.95, and one was questionable with a value of 0.62.

### Car reduction

Behavioural beliefs were best represented by a four-factor solution, consisting of disadvantages (e.g., require more planning), environmental benefits (e.g., help the environment), negative feelings (e.g., annoyed) and health benefits (e.g., make me feel healthy). Two-factor solutions were found for both normative beliefs and habit beliefs. Normative beliefs were grouped into positive role models (e.g., climate activists) and negative role models (e.g., wealthy people). Habit beliefs consisted of experience with car reduction, a factor containing both positive experience and circumstances in which people reduce their car use automatically, and negative experience (e.g., hard and unreliable). A three-factor solution was found for control beliefs, as three eigenvalues were found to be higher than 1. The solution includes facilitators (e.g., reduced price of public transport) and two types of barriers – poor alternatives (e.g., lack of reliable public transport) and practical difficulties (e.g., work being too far). A unifactorial solution best described moral norm beliefs, comprising of both positive and negative views about moral responsibility and values (e.g., I believe I have a responsibility to reduce my car use to protect the environment). Alpha reliabilities were between 0.73 and 0.92 for most factors, most being acceptable to good values, with a few excellent ones. A poor alpha reliability value of 0.52 was found for one of the control beliefs factors. The factor was retained despite its lower reliability coefficient due to its theoretical significance, as it contains beliefs about practical difficulties associated with reducing car use.

### Flight reduction

Three-factor solutions were chosen for behavioural and control beliefs. The three behavioural beliefs factors were negative personal impact (e.g., affect my mental health), environmental benefits (e.g., reduce carbon footprint) and inconvenience (e.g., be inconvenient). Barriers (e.g., worse alternatives), facilitators (e.g., efficient alternatives), and work (e.g., work circumstances changing) were the three factors representing control beliefs. A four-factor solution best represented the normative beliefs: negative role models (e.g., famous people), positive role models (e.g., climate activists), approvers (e.g., employers) and eco-approvers (e.g., people who are concerned about the environment). Unifactorial solutions were chosen for habit and moral norm beliefs. Habit beliefs were represented by individual experience with the target behaviour (e.g., I found myself automatically choosing to reduce my flights in work situations). The moral beliefs factor contained both positive and negative beliefs about moral responsibility (e.g., I believe I have a responsibility to reduce flights to protect the environment). Alpha reliabilities ranged from 0.61 to 0.96, containing acceptable, good, and excellent values.

### Regression models

Multiple regression models were used to explain: (i) the predictive capacity of belief factors (i.e., behavioural beliefs, normative beliefs, control beliefs, habit beliefs, and moral norm beliefs) on the components of the TPB model — namely, attitudes, subjective norms, and perceived behavioural control, as well as the extended components, habit and moral norms; (ii) the influence of the standard TPB variables (i.e., attitudes, subjective norms, perceived behavioural control) on intention, (iii) the role of the extended TPB model, incorporating both habit and moral norms, in predicting intention, and (iv) how intention predicts behaviour. Demographic variables (i.e., sex, ethnicity, education, and age) were controlled in all models.

### Predicting TPB variables from belief factors

Table 2 shows how attitudes, subjective norms, perceived behavioural control, habit, and moral norms were predicted by behavioural, normative, control, habit, and moral norm beliefs. Most behavioural belief factors were strong significant predictors of attitude across the five behaviours, with the proportion of variance explained being between 45% to 67%. Some of the beliefs with strong independent contributions across different behaviours were beliefs about advantages, disadvantages, positive experience, negative feelings, and health benefits associated with the target behaviour. Most habit beliefs significantly predicted habit across the five behaviours, explaining from 32% to 55% of the variance in habit. The only non-significant predictor found was negative experience for car reduction. All moral norm beliefs significantly predicted moral norms, with 33% to 58% variance explained across the target behaviours.

**Table 2:** Predicting TPB variables from belief factors.

Behaviour	Attitude			Subjective norm			Perceived behavioural control			Habit			Moral norm		
	Behavioural beliefs	Beta	R^2^	Normative beliefs	Beta	R^2^	Control beliefs	Beta	R^2^	Habit beliefs	Beta	R^2^	Moral norm beliefs	Beta	R^2^
**Sustainable transportation**	Advantages	0.65*	0.67	Young approvers	0.25*	0.04	Facilitators	0.40*	0.22	Experience	0.94*	0.46	Responsibility	0.81*	0.58
	Disadvantages	0.23*		Professional approvers	−0.05*		Barriers	0.57*							
				Disapprovers		0.14									
**Public transportation**	Disadvantages	0.39*	0.58	Approvers	0.16	0.06	Facilitators	0.06	0.04	Experience	0.50*	0.32	Responsibility	0.61*	0.33
	Eco-benefits	0.05		Positive role models	0.04		Ineffectiveness	0.16		Automatic choice	0.71*				
		Positive experience	0.36*	Negative role models		0.20*		Inconvenience	0.25						
		Practical advantages	0.06												
**Walking and cycling**	Advantages	0.74*	0.45	Positive role models	0.50*	0.10	Facilitators	0.25	0.08	Automatic choice	0.31*	0.54	Responsibility	0.73*	0.34
	Disadvantages	0.24*		Approvers	0.12		Personal barriers	−0.23		Positive experience	0.56*				
				Disapprovers	0.08		External barriers	0.36*		Negative experience	0.39*				
				Negative role models		0.04									
**Reducing car use**	Disadvantages	0.20	0.48	Positive role models	0.12	0.05	Poor alternatives	0.12	0.03	Experience	1.03*	0.55	Responsibility	0.77*	0.42
	Eco-benefits	0.15*		Negative role models	0.03		Facilitators	0.16		Negative experience	0.07				
		Negative feelings	0.20*					Practical difficulties	0.14						
		Health benefits	0.30*												
**Reducing flights**	Negative personal impact	0.15*	0.55	Negative role models	−0.05	0.13	Facilitators	0.29*	0.27	Experience	0.83*	0.48	Responsibility	0.93*	0.47
	Eco-benefits	0.37*		Approvers	0.29*		Work circumstances	−0.21							
	Inconvenience	0.31*		Eco-approvers	−0.17*		Worse alternatives	0.36*							
				Positive role models		0.07									

* *p* < 0.05

Stronger approval from young people and professionals was a significant predictor of subjective norms for sustainable transportation. Negative role models significantly predicted subjective norms for public transportation, and positive role models significantly predicted subjective norms for walking and cycling. Stronger approval from eco-activists and people who care about the environment was negatively associated with subjective norms for flight reduction. Normative beliefs explained a low proportion of variance in subjective norms, ranging from 4% to 13%. They were not significant predictors of subjective norms for car reduction.

Facilitators and barriers showed significant and positive contributions to predictions of perceived behavioural control for sustainable transportation, explaining 22% of the variance. For walking and cycling, only external barriers had a significant impact on perceived behavioural control, with only 8% of the total variance in perceived behavioural control explained. For flight reduction, 27% of the variance in perceived behavioural control was explained, and the beliefs with significant contributions addressed facilitators and worse alternatives. Control beliefs did not significantly predict perceived behavioural control for public transportation and car reduction.

### Predicting intention from TPB variables

Table 3 shows the results of the hierarchical regression analysis, displaying in the standard TPB model how the standard TPB variables predict intention. The extended TPB model broadens this analysis by incorporating two additional variables: habit and moral norm. This allows for an examination of how the inclusion of these extended TPB components further influences the prediction of behavioural intention.

**Table 3:** Predicting intention from TPB variables.

	Behaviour				
Variables	Sustainable transportation	Public Transportation	Walking and Cycling	Reducing Car Use	Reducing Flights
**Model 1**	**Beta**	**Beta**	**Beta**	**Beta**	**Beta**
**Attitudes**	0.47*	0.72*	0.17	0.59*	0.72*
**Subjective norms**	0.36*	0.43*	0.23*	0.36*	0.30*
**Perceived behavioural control**	0.38*	0.22*	0.67*	0.37*	0.07
**R** ^**2**^	0.77	0.61	0.66	0.71	0.58
**Model 2**	**Beta**	**Beta**	**Beta**	**Beta**	**Beta**
**Attitudes**	0.27*	0.18	−0.00	0.12	0.17
**Subjective norms**	0.03	0.07	0.04	0.05	0.05
**Perceived behavioural control**	0.01	0.05	0.27*	0.33*	0.05
**Habit**	0.43*	0.63*	0.40*	0.20*	0.47*
**Moral norms**	0.37*	0.15	0.27*	0.53*	0.35*
**R** ^**2**^	0.90	0.87	0.85	0.83	0.85

* *p* < 0.05

The standard model shows that attitudes were significant predictors of intention for all behaviours, except walking and cycling. Subjective norm was consistently found to be a significant predictor across all five behaviours. Perceived behavioural control was also a significant predictor for four of the behaviours, the only exception being flight reduction. The variance in intention explained by the standard model varied notably, ranging from 58% to 77%.

The extended model, with habit and moral norm added as predictors, shows that attitudes made a significant independent contribution only for sustainable transportation. Subjective norms did not significantly predict intention for any of the five behaviours. Perceived behavioural control was a strong predictor of intention for walking and cycling, and for car reduction. Habit was a significant predictor of intention across all five behaviours. Moral norms significantly predicted intention in four of the behaviours, excluding public transportation. The variance in intention explained by the extended model was very high, ranging from 83% to 90%.

The extended TPB model improved the predictive capacity of the standard TPB model, as it explained significantly more variance in intention for each one of the five behaviours (see Table 3).

### Predicting behaviour from intention

Regression models have been built to predict behaviour for four of the behaviours (i.e., sustainable transportation, public transportation, walking and cycling, and reducing car use) with intention as a predictor variable.

Intention significantly predicted behaviour for all behaviours. For sustainable transportation, intention accounted for 14% of the variance in behaviour. In the context of public transportation, the model explained a considerably higher variance, at 34%. For walking and cycling, and reducing car use, the models demonstrated an identical explanatory power, with 24% of the variance in each behaviour being accounted for by intention. The results are displayed in Table 4.

**Table 4:** Predicting behaviour from intention.

Behaviour	Intention	R^2^
**Sustainable transportation**	0.33*	0.14
**Public transportation**	0.40*	0.34
**Walking and cycling**	0.42*	0.24
**Reducing car use**	0.30*	0.24

* *p* < 0.05

## Discussion

### Support for study hypotheses

The present study investigated the factors underlying five sustainable behaviours related to transportation: (i) general sustainable transportation, (ii) public transportation, (iii) walking and cycling, (iv) reducing car use, (v) reducing flights. The study used an extended Theory of Planned Behaviour model, that included habit and moral norm. The aims of the study were to explore the impact of beliefs on the TPB variables, identifying key beliefs that could be targeted by intervention, the impact of the TPB variables on intention, and that of intention on behaviour, and to compare the standard TPB and extended TPB models.

The impact of beliefs was supported in most of the five behaviours investigated. For all five behaviours, behavioural beliefs were positively associated with attitudes, habit beliefs were positively associated with habit, and moral norm beliefs were positively associated with moral norms. Normative beliefs were positively associated with subjective norms in four of the behaviours, excluding car reduction. Control beliefs were positively associated with perceived behavioural control for sustainable transportation, walking and cycling, and flight reduction, but not in the other two.

When applying the standard model, TPB variables were significant predictors of intention in most cases across the five behaviours. A notable exception was that attitudes did not significantly predict intention for walking and cycling. A possible explanation might be that walking and cycling as active transportation behaviours may be chosen for various reasons, such as health and financial benefits. The multifaceted evaluation of walking and cycling could be an automatic process that does not translate into salient beliefs and strong attitudes. Mandal et al. [15] found that if people have more reasons to like walking, they walk more, and among the significant reasons they listed for liking walking were sustainability and financial reasons. In the belief elicitation phase, the findings indicate that the participants associate walking and cycling with being healthy, sustainable, and feeling happy and relaxed, but also consider some disadvantages, such as walking being time consuming, tiring, inconvenient or dangerous. Another possible explanation could be that walking and cycling were considered together as a single behaviour – active transportation. This combination may have obscured distinct attitudes specific to each activity, as walking and cycling might hold different motivational factors for individuals.

The standard TPB model explained a significant proportion of variance in intention, ranging from 58% to 77%. The addition of habit and moral norm significantly improved the fit of the model; the variance explained by this extended TPB model increased, ranging from 83% to 90%. Both habit and moral norm were significant predictors of intention across the five behaviours, with the exception of moral norm not significantly predicting public transportation. When the two additional variables were added to the model, the standard TPB variables become non-significant. Contrary to previous research [12], attitude did not have a significant independent effect on intention for four of the behaviours. This suggests that the effect of attitude on intention is not independent from moral norm and habit.

Previous research found that subjective norm was the most important factor for predicting intentions to use sustainable transport, such as public transport [39], but this was not supported by our research. Subjective norms did not make a significant independent contribution to predicting intentions in the extended model. Perceived behavioural control had a significant contribution for walking and cycling and for car reduction, but not for the other three behaviours. These results suggest that the extended TPB model adopted was appropriate and the inclusion of the additional predictors, habit and moral norms, contributed to the predictive power of the model. The addition of habit and moral norm lowered the individual contributions of the other TPB variables. A possible explanation could be that moral norms could be interpreted as a subset of attitudes. While attitudes encompass an individual’s evaluations of a behaviour, moral norms consider the ethical dimension and the alignment of the behaviour with one’s personal values and feelings of responsibility.

Ultimately, intention had a statistically significant impact on behaviour, but the variation explained in behaviour by intention was only 14% for sustainable transportation, 24% for walking and cycling and reducing car use, and 34% for public transportation. Flight reduction behaviour was not investigated as this would have been impractical with only a one-week follow-up. The lower explanatory power for sustainable transportation suggests that factors beyond intention might play a significant role, and is in alignment with the intention-behaviour gap observed in other studies [40]. This gap may be derived from external factors, as identified by the belief elicitation stage, including accessibility, economic constraints, and infrastructure.

### Research strengths and limitations

The strengths of the present research include the well-established theoretical framework used, the addition of extra predictors beyond the classic TPB model, the adequate sample size, and the measurement of behaviour with a follow-up for four of the behaviours.

The Theory of Planned Behaviour is a theoretical framework that shows good predictive power across a wide range of behaviours, including sustainable behaviours. It has good internal reliability, test-retest reliability, and predictive validity [41] and it also has the potential to be extended by integrating additional variables to increase its predictive power. The inclusion of habit and moral norm as additional predictors was motivated by findings from the literature [42,43] and the current study provided evidence supporting the importance of these variables.

The sample size was based on a power-analysis and five different samples were collected for the five behaviours. However, the samples were not representative of the population and as all the participants were from the United Kingdom, it is important to interpret the results considering the cultural and national context.

As mentioned in the introduction, many previous studies investigated beliefs underlying intention, but did not include a measure of behaviour. The present study measured behaviour one week after the administration of the main questionnaire for four of the behaviours. This was, however, not possible for flight reduction as this would have required a longer-term follow-up. On the other hand, several limitations can be associated with the follow-up. Firstly, behaviour assessment relied on self-reported measures. Self-report measures are commonly used, but they can lead to potential biases such as social desirability or memory recall. Another limitation of the follow-up is the short duration between the main questionnaire and the follow-up. A longer follow-up period or multiple follow-ups may be preferred for a more comprehensive understanding of behaviour.

### Future directions and practical applications

The limitations of the current study reveal some avenues for future research. Future research could compare different TPB models, including moderation effects and additional predictors supported by the literature. The present research supported the usefulness of an extended TPB model, containing habit and moral norm. While it may not be practical for some behaviours, future research should attempt to include objective measures for behaviour. For instance, car use could be measured using car mileage, and walking can be measured with a pedometer.

Based on the findings of this research, recommendations for interventions can be made for each of the behaviours investigated. Habit is a determinant of each one of the five behaviours, so breaking individuals’ habits and encouraging them to form more sustainable habits may be a first step in promoting sustainable transportation. Jager [44] suggests that to change a habit, three conditions need to be met: (i) the habit needs to be blocked and the situations that may activate the habit need to be removed, (ii) information on the negative consequences of the habit and the positive consequences of alternative habits needs to be available, and (iii) the alternative behaviour provides short-term positive outcomes. Possible ways to apply these recommendations to sustainable transportation could be to increase parking fees and costs associated with owning a car, implement carbon offset programs, develop more cycle lanes, offer bike-sharing programs, invest in reliable and frequent public transport services, etc. Information about the negative health, economic and environmental consequences of driving and flying, and the positive consequences of walking, cycling, and using public transportation should be a part of intervention campaigns. Ultimately, to ensure that the alternative behaviours have short-term positive outcomes, incentives could be offered for using public transportation or different cycling schemes. Additionally, to encourage new habit formation, different moments of change in people’s life can be considered. Times in which significant changes happen in someone’s life, such as changing jobs or getting married, may be optimal for changing behaviour [45]. This could be considered by organisations, which could encourage new employees to use cycling schemes, walking schemes, and break habits related to flying for work by supporting online attendance.

As moral norms were also found to be a significant predictor of four of the five behaviours, with the exception of public transportation, interventions should target moral norms by either encouraging the formation of moral norms, if absent, or increasing the strength of the moral norms if already present. This could be achieved by raising awareness on environmental issues and targeting some of the beliefs displayed in the belief elicitation stage such as the belief that it is not someone’s responsibility to change because other people do not change, or that it is not a moral responsibility all together.

## Conclusion

The current study investigated the factors influencing five behaviours related to sustainable transportation using an extended Theory of Planned Behaviour model, where two extra predictors were added – habit and moral norms. The hypotheses were supported by the findings, as beliefs predicted the TPB constructs, the extended TPB model had a better predictive power than the standard TPB model and explained a significant proportion of the variance in intention, and intention was a significant predictor of behaviour. The addition of habit and moral norms increased the variance explained in intention by 12–27%. The most important predictors of intention were habit and moral norms, and perceived behavioural control was significant for walking and cycling and for reducing car use.

## Supporting information

S1 File Information Sheet and Consent. (DOCX)

S2 File Questionnaires. (DOCX)

S3 File Correlation Matrices. (DOCX)

S4 File Factor Analyses. (DOCX)
