# Supplementary material for: On the road to sustainability: Applying an extended Theory of Planned Behaviour model to energy-saving transportation practices
Source: PLoS One. 2025 Jun 3;20(6):e0325196. doi: 10.1371/journal.pone.0325196 (PMC12132967; doi:10.1371/journal.pone.0325196)
Supplement: S1 File — (DOCX) [file pone.0325196.s001.docx]

**S1 File**: Information Sheet and Consent

1. Elicitation Phase

Project Title

Applying an extended Theory of Planned Behaviour model to energy-saving transportation practices

What this research will involve

This is an invitation to take part in a research study investigating sustainable transportation practices. The study consists of a questionnaire that will take approximately 5 minutes to complete. You will be asked questions related to your transportation practices. You will be compensated for your time via Prolific.

Deciding to take part

Your participation in this study is completely voluntary. If you decide not to participate, or choose to withdraw part-way through or after completion, there will be no negative consequences for you. You can discontinue or withdraw from this study at any time by closing the window, without giving a reason. Whilst you can withdraw from any current or future data collection, once you have submitted the questionnaire the data cannot be removed as it is anonymised.

Confidentiality

Your participation in this study will not be disclosed to anyone. Your identity will remain completely anonymous and will not at any point be referred to by any identifying information. Your name or any other personal details will not be recorded in any part of this questionnaire. After the project has finished, the data will be uploaded on the Open Science Framework, but any identifiable information will be anonymised.

Further information

This research has been approved by the University of Sheffield ethics committee. The University is responsible for looking after your information and using it appropriately. If you wish to make a query or file a complaint, please email the student conducting this research (abciocirlan1@sheffield.ac.uk) or her supervisor Professor Richard Rowe (r.rowe@sheffield.ac.uk). If you wish to take your complaint further, please contact the Head of the Psychology Department, Professor Elizabeth Milne (e.milne@sheffield.ac.uk).

Please tick this button to confirm you have read and understood the information provided above and agree to take part in this study.



1. Main questionnaire and follow-up

Project Title

Your views on transport and sustainability

What this research will involve

This is an invitation to take part in a research study investigating sustainable transportation practices. The study consists of a questionnaire that will take approximately 6 minutes to complete and a 1 minute follow up questionnaire after 1 week. You will be asked questions related to your transportation practices. You will be compensated for your time via Prolific.

Deciding to take part

Your participation in this study is completely voluntary. If you decide not to participate, or choose to withdraw part-way through or after completion, there will be no negative consequences for you. You can discontinue or withdraw from this study at any time by closing the window, without giving a reason. Whilst you can withdraw from any current or future data collection, once you have submited the questionnaire the data cannot be removed as it is anonymised.

Confidentiality

Your participation in this study will not be disclosed to anyone. Your identity will remain completely anonymous and will not at any point be referred to by any identifying information. Your name or any other personal details will not be recorded in any part of this questionnaire. After the project has finished, the data will be uploaded on the Open Science Framework, but any identifiable information will be removed.

Further information

This research has been approved by the University of Sheffield ethics committee. The University is responsible for looking after your information and using it appropriately. If you wish to make a query or file a complaint, please email the student conducting this research (abciocirlan1@sheffield.ac.uk) or her supervisor Professor Richard Rowe (r.rowe@sheffield.ac.uk). If you wish to take your complaint further, please contact the Head of the Psychology Department, Professor Elizabeth Milne (e.milne@sheffield.ac.uk).

Please tick this button to confirm you have read and understood the information provided above and agree to take part in this study.


