# Supplementary material for: On the road to sustainability: Applying an extended Theory of Planned Behaviour model to energy-saving transportation practices
Source: PLoS One. 2025 Jun 3;20(6):e0325196. doi: 10.1371/journal.pone.0325196 (PMC12132967; doi:10.1371/journal.pone.0325196)
Supplement: S2 File — (DOCX) [file pone.0325196.s002.docx]

**S2 File:** Questionnaires

Belief elicitation phase example

- What are the advantages for you of choosing sustainable transportation on a regular basis?
- What are the disadvantages for you of choosing sustainable transportation on a regular basis?
- What positive feelings do you associate with choosing sustainable transportation on a regular basis?
- What negative feelings do you associate with choosing sustainable transportation on a regular basis?
- Please list the individuals or groups who would think you should choose sustainable transportation on a regular basis.
- Please list the individuals or groups who would think you should not choose sustainable transportation on a regular basis.
- Sometimes, when we are not sure what to do, we look to see what others are doing. Please list the individuals or groups who are most likely to choose sustainable transportation on a regular basis. Please list the individuals or groups who are least likely to choose sustainable transportation on a regular basis.
- Please list any factors or circumstances that would make it easier for you to choose sustainable transportation on a regular basis.
- Please list any factors or circumstances that would make it difficult or prevent you from choosing sustainable transportation on a regular basis.
- Have you ever tried to choose sustainable transportation options on a regular basis? If so, what was that experience like for you? If not, what has stopped you from trying?
- What are some situations or circumstances when you find yourself automatically choosing sustainable transportation options?
- Do you believe it is a moral responsibility to engage in sustainable transportation on a regular basis? If so, why? If not, why not?
- How do your personal values influence your decision to choose sustainable transportation on a regular basis?

Main TPB questionnaire example

Please enter your Prolific ID below:

________________________________________________________________

What is your ethnicity?

- White
- Black or African American
- American Indian or Alaska Native
- Asian
- Native Hawaiian or Pacific Islander
- Other

What is the highest degree or level of school you have completed?

- Less than high school
- High school or equivalent
- Bachelor's degree (e.g., BA, BSc)
- Master's degree (e.g., MA, MSc)
- Doctorate (e.g., PhD)
- Other (please specify): __________________________________________________

**End of Block: demographic info**

**Start of Block: beh_belief**

Choosing sustainable transportation would...

|  | Strongly disagree | Disagree | Somewhat disagree | Neither agree nor disagree | Somewhat agree | Agree | Strongly agree |
| --- | --- | --- | --- | --- | --- | --- | --- |
| ...help the environment. |  |  |  |  |  |  |  |
| ...reduce my carbon footprint. |  |  |  |  |  |  |  |
| ...lead to less air pollution. |  |  |  |  |  |  |  |
| ...lower my transportation costs. |  |  |  |  |  |  |  |
| ...improve my mental health. |  |  |  |  |  |  |  |
| ...give me a sense of intrinsic reward. |  |  |  |  |  |  |  |
| ...make me happy. |  |  |  |  |  |  |  |
| ...make me feel eco-friendly. |  |  |  |  |  |  |  |
| ...make me feel morally good. |  |  |  |  |  |  |  |
| ...be inconvenient. |  |  |  |  |  |  |  |
| ...be expensive. |  |  |  |  |  |  |  |
| ...be unreliable. |  |  |  |  |  |  |  |
| ...be inaccesible. |  |  |  |  |  |  |  |
| ...be time consuming. |  |  |  |  |  |  |  |
| ...make me annoyed. |  |  |  |  |  |  |  |
| ...make me worried. |  |  |  |  |  |  |  |

**End of Block: beh_belief**

**Start of Block: norm_belief**

The following individuals or groups think I should choose sustainable transportation on a regular basis:

|  | Strongly disagree | Disagree | Somewhat disagree | Neither agree nor disagree | Somewhat agree | Agree | Strongly agree |
| --- | --- | --- | --- | --- | --- | --- | --- |
| Environmentalists |  |  |  |  |  |  |  |
| Government representatives |  |  |  |  |  |  |  |
| General public |  |  |  |  |  |  |  |
| Young people |  |  |  |  |  |  |  |
| Students |  |  |  |  |  |  |  |
| Commuters |  |  |  |  |  |  |  |
| Businessmen |  |  |  |  |  |  |  |

The following people or groups of people think I should NOT choose sustainable transportation on a regular basis:

|  | Strongly disagree | Disagree | Somewhat disagree | Neither agree nor disagree | Somewhat agree | Agree | Strongly agree |
| --- | --- | --- | --- | --- | --- | --- | --- |
| Workers |  |  |  |  |  |  |  |
| The motor industry |  |  |  |  |  |  |  |
| Non-environmentalists |  |  |  |  |  |  |  |

The following people or groups of people are most likely to use sustainable transportation on a regular basis:

|  | Strongly disagree | Disagree | Somewhat disagree | Neither agree nor disagree | Somewhat agree | Agree | Strongly agree |
| --- | --- | --- | --- | --- | --- | --- | --- |
| Young people |  |  |  |  |  |  |  |
| Climate activists |  |  |  |  |  |  |  |
| People concerned about the environment |  |  |  |  |  |  |  |

The following people or groups of people are least likely to use sustainable transportation on a regular basis:

|  | Strongly disagree | Disagree | Somewhat disagree | Neither agree nor disagree | Somewhat agree | Agree | Strongly agree |
| --- | --- | --- | --- | --- | --- | --- | --- |
| Eldery |  |  |  |  |  |  |  |
| Wealthy |  |  |  |  |  |  |  |
| People who live in isolated areas |  |  |  |  |  |  |  |

**End of Block: norm_belief**

**Start of Block: cont_belief**

The following factors or circumstances would make it easier for me to choose sustainable transportation:

|  | Strongly disagree | Disagree | Somewhat disagree | Neither agree nor disagree | Somewhat agree | Agree | Strongly agree |
| --- | --- | --- | --- | --- | --- | --- | --- |
| Increased availability |  |  |  |  |  |  |  |
| Increased efficiency |  |  |  |  |  |  |  |
| Reduced cost |  |  |  |  |  |  |  |
| Increased accesibility |  |  |  |  |  |  |  |

The following factors or circumstances would make it harder for me to choose sustainable transportation:

|  | Strongly disagree | Disagree | Somewhat disagree | Neither agree nor disagree | Somewhat agree | Agree | Strongly agree |
| --- | --- | --- | --- | --- | --- | --- | --- |
| Timings |  |  |  |  |  |  |  |
| Unreliability |  |  |  |  |  |  |  |
| Inaccesibility |  |  |  |  |  |  |  |
| Inconvenience |  |  |  |  |  |  |  |
| Price |  |  |  |  |  |  |  |

**End of Block: cont_belief**

**Start of Block: hab_belief**

Thinking about your behaviour over the last few years, how much do you agree with the following statements:

|  | Strongly disagree | Disagree | Somewhat disagree | Neither agree nor disagree | Somewhat agree | Agree | Strongly agree |
| --- | --- | --- | --- | --- | --- | --- | --- |
| I tried sustainable transportation and it was a pleasant experience. |  |  |  |  |  |  |  |
| I tried sustainable transportation when it was practical. |  |  |  |  |  |  |  |
| I tried sustainable transportation but it was difficult. |  |  |  |  |  |  |  |
| I have not tried sustainable transportation because I pick the cheapest and most convenient option. |  |  |  |  |  |  |  |

I found myself automatically choosing sustainable transportation when...

|  | Strongly disagree | Disagree | Somewhat disagree | Neither agree nor disagree | Somewhat agree | Agree | Strongly agree |
| --- | --- | --- | --- | --- | --- | --- | --- |
| I was travelling long distances. |  |  |  |  |  |  |  |
| It was most convenient. |  |  |  |  |  |  |  |
| I was going to work. |  |  |  |  |  |  |  |

**End of Block: hab_belief**

**Start of Block: moral_belief**

Thinking about individual responsibility to choose sustainable transportation, how much do you agree with the following statements:

|  | Strongly disagree | Disagree | Somewhat disagree | Neither agree nor disagree | Somewhat agree | Agree | Strongly agree |
| --- | --- | --- | --- | --- | --- | --- | --- |
| I believe we have a responsibility to choose sustainable transportation to protect the environment. |  |  |  |  |  |  |  |
| I believe we have a moral responsibility to choose sustainable transportation. |  |  |  |  |  |  |  |
| I don’t think the responsibility to choose sustainable transportation is on me. |  |  |  |  |  |  |  |

Thinking about how individual values influence the decision to choose sustainable transportation, how much do you agree with the following statements:

|  | Strongly disagree | Disagree | Somewhat disagree | Neither agree nor disagree | Somewhat agree | Agree | Strongly agree |
| --- | --- | --- | --- | --- | --- | --- | --- |
| I want to use sustainable transportation because I care for the environment. |  |  |  |  |  |  |  |
| My values do not have an impact on my decision to choose sustainable transportation. |  |  |  |  |  |  |  |

**End of Block: moral_belief**

**Start of Block: TPB**

Choosing sustainable transportation on a regular basis would be:

|  | Extremely unpleasant | Very unpleasant | Moderately unpleasant | Neither pleasant nor unpleasant | Moderately pleasant | Very pleasant | Extremely pleasant |
| --- | --- | --- | --- | --- | --- | --- | --- |
| 1 |  |  |  |  |  |  |  |

Choosing sustainable transportation on a regular basis would be:

|  | Extremely unhealthy | Very unhealthy | Moderately unhealthy | Neither healthy nor unhealthy | Moderately healthy | Very healthy | Extremely healthy |
| --- | --- | --- | --- | --- | --- | --- | --- |
| 1 |  |  |  |  |  |  |  |

Choosing sustainable transportation on a regular basis would be:

|  | Extremely detrimental | Very detrimental | Moderately detrimental | Neither detrimental nor beneficial | Moderately beneficial | Very beneficial | Extremely beneficial |
| --- | --- | --- | --- | --- | --- | --- | --- |
| 1 |  |  |  |  |  |  |  |

It is valuable for me to choose sustainable transportation on a regular basis.

|  | Strongly disagree | Disagree | Somewhat disagree | Neither agree nor disagree | Somewhat agree | Agree | Strongly agree |
| --- | --- | --- | --- | --- | --- | --- | --- |
| 1 |  |  |  |  |  |  |  |

Choosing sustainable transportation on a regular basis is a wise decision.

|  | Strongly disagree | Disagree | Somewhat disagree | Neither agree nor disagree | Somewhat agree | Agree | Strongly agree |
| --- | --- | --- | --- | --- | --- | --- | --- |
| 1 |  |  |  |  |  |  |  |

Most people who are important to me approve of me choosing sustainable transportation on a regular basis.

|  | Strongly disagree | Disagree | Somewhat disagree | Neither agree nor disagree | Somewhat agree | Agree | Strongly agree |
| --- | --- | --- | --- | --- | --- | --- | --- |
|  |  |  |  |  |  |  |  |

Most people would like it if I chose sustainable transportation on a regular basis.

|  | Strongly disagree | Disagree | Somewhat disagree | Neither agree nor disagree | Somewhat agree | Agree | Strongly agree |
| --- | --- | --- | --- | --- | --- | --- | --- |
|  |  |  |  |  |  |  |  |

My friends influence me to choose sustainable transportation on a regular basis.

|  | Strongly disagree | Disagree | Somewhat disagree | Neither agree nor disagree | Somewhat agree | Agree | Strongly agree |
| --- | --- | --- | --- | --- | --- | --- | --- |
|  |  |  |  |  |  |  |  |

My family supports me to choose sustainable transportation on a regular basis.

|  | Strongly disagree | Disagree | Somewhat disagree | Neither agree nor disagree | Somewhat agree | Agree | Strongly agree |
| --- | --- | --- | --- | --- | --- | --- | --- |
|  |  |  |  |  |  |  |  |

I am confident I can choose sustainable transportation on a regular basis.

|  | Strongly disagree | Disagree | Somewhat disagree | Neither agree nor disagree | Somewhat agree | Agree | Strongly agree |
| --- | --- | --- | --- | --- | --- | --- | --- |
|  |  |  |  |  |  |  |  |

Choosing sustainable transportation on a regular basis is up to me.

|  | Strongly disagree | Disagree | Somewhat disagree | Neither agree nor disagree | Somewhat agree | Agree | Strongly agree |
| --- | --- | --- | --- | --- | --- | --- | --- |
|  |  |  |  |  |  |  |  |

It is easy to choose sustainable transportation on a general basis.

|  | Strongly disagree | Disagree | Somewhat disagree | Neither agree nor disagree | Somewhat agree | Agree | Strongly agree |
| --- | --- | --- | --- | --- | --- | --- | --- |
|  |  |  |  |  |  |  |  |

I believe I am capable of choosing sustainable transportation on a regular basis.

|  | Strongly disagree | Disagree | Somewhat disagree | Neither agree nor disagree | Somewhat agree | Agree | Strongly agree |
| --- | --- | --- | --- | --- | --- | --- | --- |
|  |  |  |  |  |  |  |  |

**End of Block: TPB**

**Start of Block: int, habit and moral norm**

I intend to choose sustainable transportation on a regular basis.

|  | Strongly disagree | Disagree | Somewhat disagree | Neither agree nor disagree | Somewhat agree | Agree | Strongly agree |
| --- | --- | --- | --- | --- | --- | --- | --- |
|  |  |  |  |  |  |  |  |

I would like to choose sustainable transportation on a regular basis.

|  | Strongly disagree | Disagree | Somewhat disagree | Neither agree nor disagree | Somewhat agree | Agree | Strongly agree |
| --- | --- | --- | --- | --- | --- | --- | --- |
|  |  |  |  |  |  |  |  |

I plan to choose sustainable transportation on a regular basis.

|  | Strongly disagree | Disagree | Somewhat disagree | Neither agree nor disagree | Somewhat agree | Agree | Strongly agree |
| --- | --- | --- | --- | --- | --- | --- | --- |
|  |  |  |  |  |  |  |  |

I expect to choose sustainable transportation on a regular basis.

|  | Strongly disagree | Disagree | Somewhat disagree | Neither agree nor disagree | Somewhat agree | Agree | Strongly agree |
| --- | --- | --- | --- | --- | --- | --- | --- |
|  |  |  |  |  |  |  |  |

Choosing sustainable transportation has become my natural choice.

|  | Strongly disagree | Disagree | Somewhat disagree | Neither agree nor disagree | Somewhat agree | Agree | Strongly agree |
| --- | --- | --- | --- | --- | --- | --- | --- |
|  |  |  |  |  |  |  |  |

I frequently choose sustainable transportation.

|  | Strongly disagree | Disagree | Somewhat disagree | Neither agree nor disagree | Somewhat agree | Agree | Strongly agree |
| --- | --- | --- | --- | --- | --- | --- | --- |
|  |  |  |  |  |  |  |  |

It would feel right for me to opt for sustainable transportation on a general basis.

|  | Strongly disagree | Disagree | Somewhat disagree | Neither agree nor disagree | Somewhat agree | Agree | Strongly agree |
| --- | --- | --- | --- | --- | --- | --- | --- |
|  |  |  |  |  |  |  |  |

I would feel guilty if I did not opt for sustainable transportation on a general basis.

|  | Strongly disagree | Disagree | Somewhat disagree | Neither agree nor disagree | Somewhat agree | Agree | Strongly agree |
| --- | --- | --- | --- | --- | --- | --- | --- |
|  |  |  |  |  |  |  |  |

**End of Block: int, habit and moral norm**

**Start of Block: past_behaviour**

In the last week I have chosen sustainable transportation...

- Never
- Occassionally
- Often
- Very often
- Always
