# Supplementary material for: On the road to sustainability: Applying an extended Theory of Planned Behaviour model to energy-saving transportation practices
Source: PLoS One. 2025 Jun 3;20(6):e0325196. doi: 10.1371/journal.pone.0325196 (PMC12132967; doi:10.1371/journal.pone.0325196)
Supplement: S3 File — (DOCX) [file pone.0325196.s003.docx]

**S3 File:** Correlation Matrices (raw data)

Sustainable Transportation

- Behavioural Beliefs

beh_bel1 1.0000000 0.8243271 0.7069462 0.3933497 0.5593121 0.6364094

beh_bel2 0.8243271 1.0000000 0.6719317 0.4255190 0.5125471 0.5303673

beh_bel3 0.7069462 0.6719317 1.0000000 0.2585818 0.3951428 0.4183654

beh_bel4 0.3933497 0.4255190 0.2585818 1.0000000 0.6727499 0.4988966

beh_bel5 0.5593121 0.5125471 0.3951428 0.6727499 1.0000000 0.7583475

beh_bel6 0.6364094 0.5303673 0.4183654 0.4988966 0.7583475 1.0000000

beh_bel7 0.5888377 0.5203815 0.4234760 0.5330564 0.6978773 0.8141611

beh_bel8 0.7681362 0.7640473 0.5954606 0.5191579 0.6052091 0.7370522

beh_bel9 0.6987465 0.7059732 0.5460024 0.4528905 0.5843559 0.7140310

beh_bel10 0.3284098 0.3494437 0.3114918 0.2374387 0.3546012 0.3858887

beh_bel11 0.2102500 0.1741960 0.2529154 0.5528512 0.4837559 0.2948232

beh_bel12 0.4359916 0.3495376 0.2652012 0.3230103 0.3969677 0.4221082

beh_bel13 0.3812464 0.3854597 0.2905407 0.4404605 0.4570394 0.3949642

beh_bel14 0.3979579 0.3909044 0.3106960 0.4180834 0.4269830 0.4520365

beh_bel15 0.5157702 0.4556004 0.4145426 0.3364683 0.4053032 0.4525371

beh_bel16 0.4836670 0.4596884 0.3942019 0.2721521 0.4374395 0.4074727

beh_bel7 beh_bel8 beh_bel9 beh_bel10 beh_bel11 beh_bel12

beh_bel1 0.5888377 0.7681362 0.6987465 0.3284098 0.2102500 0.4359916

beh_bel2 0.5203815 0.7640473 0.7059732 0.3494437 0.1741960 0.3495376

beh_bel3 0.4234760 0.5954606 0.5460024 0.3114918 0.2529154 0.2652012

beh_bel4 0.5330564 0.5191579 0.4528905 0.2374387 0.5528512 0.3230103

beh_bel5 0.6978773 0.6052091 0.5843559 0.3546012 0.4837559 0.3969677

beh_bel6 0.8141611 0.7370522 0.7140310 0.3858887 0.2948232 0.4221082

beh_bel7 1.0000000 0.6620385 0.6496332 0.4899307 0.3952033 0.5216466

beh_bel8 0.6620385 1.0000000 0.8870165 0.3991408 0.2823949 0.4172001

beh_bel9 0.6496332 0.8870165 1.0000000 0.4228637 0.3334233 0.4549171

beh_bel10 0.4899307 0.3991408 0.4228637 1.0000000 0.3410763 0.5780773

beh_bel11 0.3952033 0.2823949 0.3334233 0.3410763 1.0000000 0.4445097

beh_bel12 0.5216466 0.4172001 0.4549171 0.5780773 0.4445097 1.0000000

beh_bel13 0.4955456 0.4068849 0.4605703 0.5720583 0.5794147 0.7002431

beh_bel14 0.5925199 0.4510300 0.4715787 0.7026890 0.4281550 0.6792393

beh_bel15 0.6606637 0.4943320 0.5458662 0.6329569 0.3732012 0.6628742

beh_bel16 0.5326453 0.3993663 0.4539246 0.5668879 0.3382875 0.5745521

beh_bel13 beh_bel14 beh_bel15 beh_bel16

beh_bel1 0.3812464 0.3979579 0.5157702 0.4836670

beh_bel2 0.3854597 0.3909044 0.4556004 0.4596884

beh_bel3 0.2905407 0.3106960 0.4145426 0.3942019

beh_bel4 0.4404605 0.4180834 0.3364683 0.2721521

beh_bel5 0.4570394 0.4269830 0.4053032 0.4374395

beh_bel6 0.3949642 0.4520365 0.4525371 0.4074727

beh_bel7 0.4955456 0.5925199 0.6606637 0.5326453

beh_bel8 0.4068849 0.4510300 0.4943320 0.3993663

beh_bel9 0.4605703 0.4715787 0.5458662 0.4539246

beh_bel10 0.5720583 0.7026890 0.6329569 0.5668879

beh_bel11 0.5794147 0.4281550 0.3732012 0.3382875

beh_bel12 0.7002431 0.6792393 0.6628742 0.5745521

beh_bel13 1.0000000 0.6194628 0.6324980 0.5844314

beh_bel14 0.6194628 1.0000000 0.7638461 0.6059098

beh_bel15 0.6324980 0.7638461 1.0000000 0.7393920

beh_bel16 0.5844314 0.6059098 0.7393920 1.0000000

- Normative Beliefs

inj_pos_1 inj_pos_2 inj_pos_3 inj_pos_4 inj_pos_5

inj_pos_1 1.00000000 0.09372043 0.05325554 0.20249762 0.25911067

inj_pos_2 0.09372043 1.00000000 0.43374562 0.28939711 0.31406021

inj_pos_3 0.05325554 0.43374562 1.00000000 0.68253542 0.58457779

inj_pos_4 0.20249762 0.28939711 0.68253542 1.00000000 0.89758177

inj_pos_5 0.25911067 0.31406021 0.58457779 0.89758177 1.00000000

inj_pos_6 0.06693461 0.42712984 0.63914736 0.56668185 0.56281559

inj_pos_7 -0.01660416 0.56193730 0.69643223 0.46359344 0.38597786

inj_neg_1 0.29577329 0.15819529 0.32332261 0.14121439 0.16631752

inj_neg_2 -0.04829676 0.32379668 0.37512861 0.20485720 0.16036807

inj_neg_3 -0.08469061 0.21786228 0.28887061 0.06990387 0.03184942

desc_pos_1 0.21174075 0.04412248 0.30135989 0.58029393 0.52023697

desc_pos_2 0.17992094 -0.07150790 0.18135241 0.19682756 0.19180280

desc_pos_3 0.32806537 0.01277391 0.18438373 0.22498058 0.20957052

desc_neg_1 -0.02038096 -0.32013455 -0.07179415 -0.17248779 -0.16648570

desc_neg_2 -0.17213790 -0.26338406 -0.23884744 -0.23498774 -0.28787612

desc_neg_3 -0.16481139 -0.14146180 -0.10874518 -0.12970814 -0.11599643

inj_pos_6 inj_pos_7 inj_neg_1 inj_neg_2 inj_neg_3

inj_pos_1 0.06693461 -0.01660416 0.295773291 -0.04829676 -0.084690614

inj_pos_2 0.42712984 0.56193730 0.158195293 0.32379668 0.217862278

inj_pos_3 0.63914736 0.69643223 0.323322606 0.37512861 0.288870609

inj_pos_4 0.56668185 0.46359344 0.141214389 0.20485720 0.069903871

inj_pos_5 0.56281559 0.38597786 0.166317525 0.16036807 0.031849418

inj_pos_6 1.00000000 0.63063052 0.192220779 0.33880469 0.205132562

inj_pos_7 0.63063052 1.00000000 0.254944130 0.55298710 0.442223647

inj_neg_1 0.19222078 0.25494413 1.000000000 0.53598040 0.493027014

inj_neg_2 0.33880469 0.55298710 0.535980404 1.00000000 0.710624122

inj_neg_3 0.20513256 0.44222365 0.493027014 0.71062412 1.000000000

desc_pos_1 0.28532317 0.09938579 -0.041785149 -0.08612121 -0.152799276

desc_pos_2 0.09816766 -0.04885853 0.043889106 -0.01268466 -0.003350034

desc_pos_3 0.23073329 -0.04593276 0.245287240 0.01377801 0.038292462

desc_neg_1 -0.16368028 -0.11285932 -0.019309330 -0.19899098 -0.103914102

desc_neg_2 -0.11392195 -0.11840970 0.003318896 0.21192147 0.221301089

desc_neg_3 -0.03363465 0.09039842 -0.074218357 0.10203727 0.026016703

desc_pos_1 desc_pos_2 desc_pos_3 desc_neg_1 desc_neg_2

inj_pos_1 0.21174075 0.179920940 0.32806537 -0.02038096 -0.172137900

inj_pos_2 0.04412248 -0.071507898 0.01277391 -0.32013455 -0.263384057

inj_pos_3 0.30135989 0.181352413 0.18438373 -0.07179415 -0.238847439

inj_pos_4 0.58029393 0.196827561 0.22498058 -0.17248779 -0.234987735

inj_pos_5 0.52023697 0.191802804 0.20957052 -0.16648570 -0.287876117

inj_pos_6 0.28532317 0.098167656 0.23073329 -0.16368028 -0.113921955

inj_pos_7 0.09938579 -0.048858534 -0.04593276 -0.11285932 -0.118409701

inj_neg_1 -0.04178515 0.043889106 0.24528724 -0.01930933 0.003318896

inj_neg_2 -0.08612121 -0.012684662 0.01377801 -0.19899098 0.211921469

inj_neg_3 -0.15279928 -0.003350034 0.03829246 -0.10391410 0.221301089

desc_pos_1 1.00000000 0.259607200 0.17623408 0.05609913 -0.220717391

desc_pos_2 0.25960720 1.000000000 0.77668558 -0.13255663 0.026369506

desc_pos_3 0.17623408 0.776685576 1.00000000 -0.23499840 0.028817421

desc_neg_1 0.05609913 -0.132556627 -0.23499840 1.00000000 -0.163006566

desc_neg_2 -0.22071739 0.026369506 0.02881742 -0.16300657 1.000000000

desc_neg_3 -0.08181937 -0.104992968 -0.17569670 0.18305821 0.479405261

desc_neg_3

inj_pos_1 -0.16481139

inj_pos_2 -0.14146180

inj_pos_3 -0.10874518

inj_pos_4 -0.12970814

inj_pos_5 -0.11599643

inj_pos_6 -0.03363465

inj_pos_7 0.09039842

inj_neg_1 -0.07421836

inj_neg_2 0.10203727

inj_neg_3 0.02601670

desc_pos_1 -0.08181937

desc_pos_2 -0.10499297

desc_pos_3 -0.17569670

desc_neg_1 0.18305821

desc_neg_2 0.47940526

desc_neg_3 1.00000000

- Control Beliefs

fac1 fac2 fac3 fac4 bar1

fac1 1.00000000 0.8056472 0.68307252 0.79607926 -0.17585497

fac2 0.80564723 1.0000000 0.68061515 0.77359784 -0.11734283

fac3 0.68307252 0.6806152 1.00000000 0.68854254 -0.02030164

fac4 0.79607926 0.7735978 0.68854254 1.00000000 -0.22793917

bar1 -0.17585497 -0.1173428 -0.02030164 -0.22793917 1.00000000

bar2 -0.25635469 -0.1779810 -0.08369359 -0.29174811 0.61366684

bar3 -0.22520918 -0.1254342 -0.02487605 -0.30548967 0.42766338

bar4 0.08811891 0.1736151 0.07287416 0.02031111 0.41830683

bar5 -0.09700416 -0.0474443 -0.27048617 -0.24565625 0.25656175

bar2 bar3 bar4 bar5

fac1 -0.25635469 -0.22520918 0.08811891 -0.09700416

fac2 -0.17798101 -0.12543424 0.17361507 -0.04744430

fac3 -0.08369359 -0.02487605 0.07287416 -0.27048617

fac4 -0.29174811 -0.30548967 0.02031111 -0.24565625

bar1 0.61366684 0.42766338 0.41830683 0.25656175

bar2 1.00000000 0.55408339 0.47450445 0.28868644

bar3 0.55408339 1.00000000 0.48162934 0.42906709

bar4 0.47450445 0.48162934 1.00000000 0.43310502

bar5 0.28868644 0.42906709 0.43310502 1.00000000

- Habit Beliefs

exp1 exp2 exp3 exp4 auto1 auto2

exp1 1.00000000 0.7301688 -0.05869706 0.42103337 0.2900740 0.4972007

exp2 0.73016881 1.0000000 -0.20888910 0.45981118 0.1927847 0.5657117

exp3 -0.05869706 -0.2088891 1.00000000 -0.09219582 -0.2500107 -0.2475029

exp4 0.42103337 0.4598112 -0.09219582 1.00000000 0.2297397 0.2217045

auto1 0.29007402 0.1927847 -0.25001067 0.22973970 1.0000000 0.3149871

auto2 0.49720073 0.5657117 -0.24750287 0.22170455 0.3149871 1.0000000

auto3 0.52749262 0.4453172 0.06460629 0.37937205 0.2594806 0.2875986

auto3

exp1 0.52749262

exp2 0.44531717

exp3 0.06460629

exp4 0.37937205

auto1 0.25948062

auto2 0.28759855

auto3 1.00000000

- Moral Norm Beliefs

resp1 resp2 resp3 val1 val2

resp1 1.0000000 0.9095103 0.6213014 0.8385240 0.5179832

resp2 0.9095103 1.0000000 0.6206369 0.8210409 0.5438983

resp3 0.6213014 0.6206369 1.0000000 0.5292178 0.5311582

val1 0.8385240 0.8210409 0.5292178 1.0000000 0.5829853

val2 0.5179832 0.5438983 0.5311582 0.5829853 1.0000000

Public Transportation

- Behavioural Beliefs

beh_bel1 beh_bel2 beh_bel3 beh_bel4 beh_bel5

beh_bel1 1.00000000 0.82132320 0.571663642 0.02359763 0.01417640

beh_bel2 0.82132320 1.00000000 0.552291587 -0.04729504 0.02440219

beh_bel3 0.57166364 0.55229159 1.000000000 0.06350021 0.05403808

beh_bel4 0.02359763 -0.04729504 0.063500212 1.00000000 0.57016947

beh_bel5 0.01417640 0.02440219 0.054038077 0.57016947 1.00000000

beh_bel6 0.37697486 0.37377870 0.388537039 0.17500336 0.24743861

beh_bel7 0.27213154 0.26913773 0.102916039 0.46894059 0.51574184

beh_bel8 0.04548418 0.01571737 -0.161675591 0.42350642 0.51470810

beh_bel9 0.22196316 0.26430385 0.147167118 0.35173152 0.39316712

beh_bel10 0.09957927 0.02387544 0.036411911 0.60633616 0.38385235

beh_bel11 0.05963737 0.06708073 -0.036456931 0.27130151 0.50576167

beh_bel12 0.25000238 0.23974169 0.063000256 0.34018303 0.27976542

beh_bel13 0.18944185 0.19123628 0.037457614 0.33014341 0.37323902

beh_bel14 0.04794814 0.07746641 -0.004113851 0.32360970 0.52922191

beh_bel15 0.12884805 0.13655290 0.008930963 0.23723320 0.41016957

beh_bel16 -0.04750091 -0.05081260 -0.156219430 0.22979227 0.31246874

beh_bel6 beh_bel7 beh_bel8 beh_bel9 beh_bel10

beh_bel1 0.37697486 0.2721315 0.04548418 0.2219632 0.09957927

beh_bel2 0.37377870 0.2691377 0.01571737 0.2643039 0.02387544

beh_bel3 0.38853704 0.1029160 -0.16167559 0.1471671 0.03641191

beh_bel4 0.17500336 0.4689406 0.42350642 0.3517315 0.60633616

beh_bel5 0.24743861 0.5157418 0.51470810 0.3931671 0.38385235

beh_bel6 1.00000000 0.4715844 0.23686902 0.3726267 0.07261461

beh_bel7 0.47158442 1.0000000 0.55143767 0.7274105 0.23808069

beh_bel8 0.23686902 0.5514377 1.00000000 0.5523371 0.27243808

beh_bel9 0.37262670 0.7274105 0.55233715 1.0000000 0.26388827

beh_bel10 0.07261461 0.2380807 0.27243808 0.2638883 1.00000000

beh_bel11 0.17885832 0.3960698 0.54780419 0.4010080 0.44251377

beh_bel12 0.25631216 0.5941951 0.49204193 0.6429673 0.44314367

beh_bel13 0.21094484 0.4817140 0.42213038 0.5982586 0.52916090

beh_bel14 0.21165567 0.3953148 0.55221389 0.5033871 0.36586845

beh_bel15 0.02876330 0.2348427 0.48211593 0.3543801 0.31298506

beh_bel16 0.20138165 0.2506171 0.44510879 0.2452491 0.42266958

beh_bel11 beh_bel12 beh_bel13 beh_bel14 beh_bel15

beh_bel1 0.05963737 0.25000238 0.18944185 0.047948140 0.128848049

beh_bel2 0.06708073 0.23974169 0.19123628 0.077466415 0.136552899

beh_bel3 -0.03645693 0.06300026 0.03745761 -0.004113851 0.008930963

beh_bel4 0.27130151 0.34018303 0.33014341 0.323609701 0.237233196

beh_bel5 0.50576167 0.27976542 0.37323902 0.529221908 0.410169568

beh_bel6 0.17885832 0.25631216 0.21094484 0.211655674 0.028763304

beh_bel7 0.39606982 0.59419507 0.48171400 0.395314823 0.234842652

beh_bel8 0.54780419 0.49204193 0.42213038 0.552213890 0.482115931

beh_bel9 0.40100801 0.64296726 0.59825865 0.503387135 0.354380122

beh_bel10 0.44251377 0.44314367 0.52916090 0.365868454 0.312985056

beh_bel11 1.00000000 0.45943663 0.52946289 0.656001211 0.566877976

beh_bel12 0.45943663 1.00000000 0.77785652 0.558835332 0.386401631

beh_bel13 0.52946289 0.77785652 1.00000000 0.600234524 0.455666233

beh_bel14 0.65600121 0.55883533 0.60023452 1.000000000 0.661042966

beh_bel15 0.56687798 0.38640163 0.45566623 0.661042966 1.000000000

beh_bel16 0.49204905 0.43495052 0.45876793 0.554878128 0.507760004

beh_bel16

beh_bel1 -0.04750091

beh_bel2 -0.05081260

beh_bel3 -0.15621943

beh_bel4 0.22979227

beh_bel5 0.31246874

beh_bel6 0.20138165

beh_bel7 0.25061712

beh_bel8 0.44510879

beh_bel9 0.24524908

beh_bel10 0.42266958

beh_bel11 0.49204905

beh_bel12 0.43495052

beh_bel13 0.45876793

beh_bel14 0.55487813

beh_bel15 0.50776000

beh_bel16 1.00000000

- Normative Beliefs

inj_pos_1 inj_pos_2 inj_pos_3 inj_pos_4 inj_pos_5

inj_pos_1 1.00000000 0.2578741580 -0.08098027 -0.00320546 0.040795258

inj_pos_2 0.25787416 1.0000000000 0.18436403 0.13414764 0.007540493

inj_pos_3 -0.08098027 0.1843640275 1.00000000 0.61639164 0.505077114

inj_pos_4 -0.00320546 0.1341476411 0.61639164 1.00000000 0.483103121

inj_pos_5 0.04079526 0.0075404925 0.50507711 0.48310312 1.000000000

inj_pos_6 0.14431272 -0.0116107070 0.26175484 0.40874915 0.483838780

inj_pos_7 0.09748271 0.0659591039 0.28134783 0.50439561 0.407810720

inj_neg_1 -0.07334293 -0.0008898417 0.14457548 0.05887492 -0.066133657

desc_pos_1 0.20332987 0.1357598655 0.06141642 -0.03942260 0.096993954

desc_pos_2 0.41565782 0.2019801796 0.05713726 0.04357174 0.191709321

desc_pos_3 0.29396628 0.2935070858 0.11093477 0.25462409 0.068623597

desc_pos_4 0.39737642 0.2967215932 0.14690774 0.19897495 0.301169102

desc_pos_5 0.01819508 0.0268144908 0.15557592 0.28662977 0.277161253

desc_pos_6 0.23195276 0.2108414611 0.05445530 0.14382372 0.241752950

desc_neg_1 -0.36468986 -0.1226356765 0.33714405 0.17587960 0.234629913

desc_neg_2 -0.19108059 -0.1031524518 0.29669566 0.04002439 0.207494380

desc_neg_3 -0.18144199 -0.0165153574 0.22182075 0.02866245 0.136502010

inj_pos_6 inj_pos_7 inj_neg_1 desc_pos_1 desc_pos_2

inj_pos_1 0.14431272 0.097482714 -0.0733429261 0.20332987 0.41565782

inj_pos_2 -0.01161071 0.065959104 -0.0008898417 0.13575987 0.20198018

inj_pos_3 0.26175484 0.281347829 0.1445754785 0.06141642 0.05713726

inj_pos_4 0.40874915 0.504395613 0.0588749206 -0.03942260 0.04357174

inj_pos_5 0.48383878 0.407810720 -0.0661336573 0.09699395 0.19170932

inj_pos_6 1.00000000 0.556637219 0.0349049744 0.05109391 0.15759468

inj_pos_7 0.55663722 1.000000000 0.0021495619 -0.03472420 0.26276117

inj_neg_1 0.03490497 0.002149562 1.0000000000 0.15083175 -0.12962396

desc_pos_1 0.05109391 -0.034724197 0.1508317501 1.00000000 0.47910160

desc_pos_2 0.15759468 0.262761172 -0.1296239572 0.47910160 1.00000000

desc_pos_3 0.17808143 0.224908232 0.0995554282 0.15126882 0.39290560

desc_pos_4 0.12324592 0.097465947 -0.0303879116 0.32325192 0.43111775

desc_pos_5 0.24540744 0.204525484 0.0087233679 -0.02617048 0.06333459

desc_pos_6 0.18306648 0.075167254 -0.0657055238 0.25576200 0.41715735

desc_neg_1 0.07798285 0.089689478 0.0956253724 -0.08939467 -0.11779187

desc_neg_2 0.05588620 0.020646143 0.1631386886 0.03536358 -0.01214062

desc_neg_3 0.02109648 0.030752872 0.3438143597 0.00328057 -0.06961617

desc_pos_3 desc_pos_4 desc_pos_5 desc_pos_6 desc_neg_1

inj_pos_1 0.29396628 0.39737642 0.018195076 0.23195276 -0.36468986

inj_pos_2 0.29350709 0.29672159 0.026814491 0.21084146 -0.12263568

inj_pos_3 0.11093477 0.14690774 0.155575920 0.05445530 0.33714405

inj_pos_4 0.25462409 0.19897495 0.286629775 0.14382372 0.17587960

inj_pos_5 0.06862360 0.30116910 0.277161253 0.24175295 0.23462991

inj_pos_6 0.17808143 0.12324592 0.245407437 0.18306648 0.07798285

inj_pos_7 0.22490823 0.09746595 0.204525484 0.07516725 0.08968948

inj_neg_1 0.09955543 -0.03038791 0.008723368 -0.06570552 0.09562537

desc_pos_1 0.15126882 0.32325192 -0.026170483 0.25576200 -0.08939467

desc_pos_2 0.39290560 0.43111775 0.063334593 0.41715735 -0.11779187

desc_pos_3 1.00000000 0.43803338 0.136645515 0.25673084 -0.07290831

desc_pos_4 0.43803338 1.00000000 0.189822450 0.39910089 -0.15337381

desc_pos_5 0.13664552 0.18982245 1.000000000 0.26890481 -0.02883466

desc_pos_6 0.25673084 0.39910089 0.268904810 1.00000000 -0.02282108

desc_neg_1 -0.07290831 -0.15337381 -0.028834660 -0.02282108 1.00000000

desc_neg_2 -0.08105379 -0.14147334 -0.061721841 -0.00860772 0.66610296

desc_neg_3 -0.06646022 -0.11769102 -0.040042499 -0.24959075 0.46526763

desc_neg_2 desc_neg_3

inj_pos_1 -0.19108059 -0.18144199

inj_pos_2 -0.10315245 -0.01651536

inj_pos_3 0.29669566 0.22182075

inj_pos_4 0.04002439 0.02866245

inj_pos_5 0.20749438 0.13650201

inj_pos_6 0.05588620 0.02109648

inj_pos_7 0.02064614 0.03075287

inj_neg_1 0.16313869 0.34381436

desc_pos_1 0.03536358 0.00328057

desc_pos_2 -0.01214062 -0.06961617

desc_pos_3 -0.08105379 -0.06646022

desc_pos_4 -0.14147334 -0.11769102

desc_pos_5 -0.06172184 -0.04004250

desc_pos_6 -0.00860772 -0.24959075

desc_neg_1 0.66610296 0.46526763

desc_neg_2 1.00000000 0.46514982

desc_neg_3 0.46514982 1.00000000

- Control Beliefs

fac1 fac2 fac3 fac4 fac5

fac1 1.00000000 0.70478973 0.40956363 0.56852501 0.37424260

fac2 0.70478973 1.00000000 0.48136803 0.62323677 0.39382998

fac3 0.40956363 0.48136803 1.00000000 0.60669598 0.64714763

fac4 0.56852501 0.62323677 0.60669598 1.00000000 0.59987532

fac5 0.37424260 0.39382998 0.64714763 0.59987532 1.00000000

fac6 0.74081329 0.73213343 0.54328590 0.69902119 0.55289134

bar1 -0.32719860 -0.29657083 -0.73325061 -0.35339018 -0.45153280

bar2 -0.25932119 -0.33525617 0.01438619 -0.21938313 -0.12097934

bar3 -0.35964725 -0.43725743 -0.06048372 -0.21666985 -0.12187706

bar4 -0.17146746 -0.32327608 -0.01754507 -0.17592489 -0.09423479

bar5 0.02799960 -0.02834411 0.07512372 -0.10086075 -0.02661101

bar6 0.04184050 0.07133526 0.01411772 -0.11970266 -0.10734662

bar7 -0.05332722 0.11876340 0.04599287 -0.04976284 -0.21425645

fac6 bar1 bar2 bar3 bar4

fac1 0.74081329 -0.3271986 -0.25932119 -0.35964725 -0.17146746

fac2 0.73213343 -0.2965708 -0.33525617 -0.43725743 -0.32327608

fac3 0.54328590 -0.7332506 0.01438619 -0.06048372 -0.01754507

fac4 0.69902119 -0.3533902 -0.21938313 -0.21666985 -0.17592489

fac5 0.55289134 -0.4515328 -0.12097934 -0.12187706 -0.09423479

fac6 1.00000000 -0.3148141 -0.28885332 -0.30190280 -0.18869607

bar1 -0.31481411 1.0000000 0.17560518 0.28687164 0.19441469

bar2 -0.28885332 0.1756052 1.00000000 0.82497990 0.64129216

bar3 -0.30190280 0.2868716 0.82497990 1.00000000 0.74477337

bar4 -0.18869607 0.1944147 0.64129216 0.74477337 1.00000000

bar5 0.02939689 0.1585362 0.43540183 0.48755158 0.61686436

bar6 0.04570597 0.1859242 0.27684342 0.33618793 0.45443429

bar7 0.04912745 0.1110819 0.09811526 0.12822105 0.07807860

bar5 bar6 bar7

fac1 0.02799960 0.04184050 -0.05332722

fac2 -0.02834411 0.07133526 0.11876340

fac3 0.07512372 0.01411772 0.04599287

fac4 -0.10086075 -0.11970266 -0.04976284

fac5 -0.02661101 -0.10734662 -0.21425645

fac6 0.02939689 0.04570597 0.04912745

bar1 0.15853619 0.18592418 0.11108194

bar2 0.43540183 0.27684342 0.09811526

bar3 0.48755158 0.33618793 0.12822105

bar4 0.61686436 0.45443429 0.07807860

bar5 1.00000000 0.76072703 0.40567699

bar6 0.76072703 1.00000000 0.45617654

bar7 0.40567699 0.45617654 1.00000000

- Habit Beliefs

exp1 exp2 exp3 exp4 exp5

exp1 1.000000000 0.3854709 0.49982878 0.47613885 0.36756289

exp2 0.385470856 1.0000000 0.15216953 0.39281203 0.29757446

exp3 0.499828784 0.1521695 1.00000000 0.34625816 0.39431788

exp4 0.476138852 0.3928120 0.34625816 1.00000000 0.55762100

exp5 0.367562885 0.2975745 0.39431788 0.55762100 1.00000000

exp6 -0.099256547 0.1703602 -0.01259592 -0.15783884 -0.22110018

hab1 -0.053757749 0.2409918 0.05738587 0.10415347 0.03122838

hab2 -0.006195409 0.3668969 -0.05709123 0.26171034 0.08291954

hab3 0.099799763 0.3326831 0.01834464 0.10881857 -0.01349827

hab4 0.085221635 0.1877801 -0.04710372 0.07526118 -0.02131004

exp6 hab1 hab2 hab3 hab4

exp1 -0.09925655 -0.05375775 -0.006195409 0.09979976 0.08522164

exp2 0.17036019 0.24099176 0.366896926 0.33268312 0.18778014

exp3 -0.01259592 0.05738587 -0.057091235 0.01834464 -0.04710372

exp4 -0.15783884 0.10415347 0.261710339 0.10881857 0.07526118

exp5 -0.22110018 0.03122838 0.082919544 -0.01349827 -0.02131004

exp6 1.00000000 0.16359895 0.070830974 0.35365272 0.09472713

hab1 0.16359895 1.00000000 0.651079496 0.50115747 0.16509019

hab2 0.07083097 0.65107950 1.000000000 0.43425088 0.12989068

hab3 0.35365272 0.50115747 0.434250883 1.00000000 0.57418015

hab4 0.09472713 0.16509019 0.129890681 0.57418015 1.00000000

- Moral Norm Beliefs

resp1 resp2 resp3 val1 val2

resp1 1.00000000 0.5698956 0.4932117 0.09307407 0.6310631

resp2 0.56989564 1.0000000 0.6031280 0.14244606 0.3834176

resp3 0.49321174 0.6031280 1.0000000 0.12116343 0.3597690

val1 0.09307407 0.1424461 0.1211634 1.00000000 0.2394576

val2 0.63106310 0.3834176 0.3597690 0.23945758 1.0000000

Walking and Cycling

- Behavioural Beliefs

beh_bel1 beh_bel2 beh_bel3 beh_bel4 beh_bel5

beh_bel1 1.00000000 0.70846202 0.26852107 0.22573517 0.41861835

beh_bel2 0.70846202 1.00000000 0.31601110 0.29932665 0.40602841

beh_bel3 0.26852107 0.31601110 1.00000000 0.17437146 0.31698860

beh_bel4 0.22573517 0.29932665 0.17437146 1.00000000 0.37610073

beh_bel5 0.41861835 0.40602841 0.31698860 0.37610073 1.00000000

beh_bel6 0.21684371 0.26583152 0.34581298 0.36031059 0.55314384

beh_bel7 0.39270408 0.39260602 0.13766221 0.54997332 0.36278915

beh_bel8 0.32149133 0.39316215 0.05758592 0.48867861 0.18038694

beh_bel9 0.49497521 0.52563609 0.23213411 0.45969493 0.28697936

beh_bel10 0.43716314 0.51376797 0.13537198 0.42465874 0.22663802

beh_bel11 0.22904098 0.38654025 0.20881527 0.42759300 0.09509579

beh_bel12 0.04669817 0.00000000 -0.02850171 0.03644583 -0.03611222

beh_bel13 0.02917488 -0.04614670 -0.13598555 0.19549537 -0.04807849

beh_bel14 -0.01414449 0.07435894 0.09901138 -0.01246899 -0.19696934

beh_bel15 0.30771632 0.32982539 0.10322452 0.01919937 0.17673198

beh_bel16 0.08413153 0.06989996 -0.04003380 0.11901609 -0.17115493

beh_bel17 0.09998679 0.06167945 -0.02669168 0.11934016 -0.18014971

beh_bel18 0.08704700 0.05746879 -0.03857963 0.04077083 -0.15781016

beh_bel6 beh_bel7 beh_bel8 beh_bel9 beh_bel10

beh_bel1 0.21684371 0.392704082 0.32149133 0.4949752 0.43716314

beh_bel2 0.26583152 0.392606019 0.39316215 0.5256361 0.51376797

beh_bel3 0.34581298 0.137662211 0.05758592 0.2321341 0.13537198

beh_bel4 0.36031059 0.549973323 0.48867861 0.4596949 0.42465874

beh_bel5 0.55314384 0.362789149 0.18038694 0.2869794 0.22663802

beh_bel6 1.00000000 0.332396988 0.33517734 0.3414389 0.53289748

beh_bel7 0.33239699 1.000000000 0.66663794 0.6204820 0.62295028

beh_bel8 0.33517734 0.666637940 1.00000000 0.7062243 0.77114509

beh_bel9 0.34143886 0.620482016 0.70622428 1.0000000 0.69399924

beh_bel10 0.53289748 0.622950278 0.77114509 0.6939992 1.00000000

beh_bel11 0.32302677 0.379250122 0.69599726 0.5652364 0.63572837

beh_bel12 -0.03102897 0.224862525 0.23238037 0.1132072 0.10355086

beh_bel13 0.09110366 -0.021943608 0.28342437 0.1266205 0.19438631

beh_bel14 -0.12892622 -0.015743658 0.24928933 0.1571723 0.09387284

beh_bel15 0.19196719 0.244539896 0.29087339 0.3081336 0.25286245

beh_bel16 0.09851494 0.212314450 0.46976906 0.3448876 0.35191177

beh_bel17 -0.22428615 -0.006618992 0.12835148 0.1049839 0.01614542

beh_bel18 0.12517798 0.169290594 0.44944527 0.3140409 0.39113653

beh_bel11 beh_bel12 beh_bel13 beh_bel14 beh_bel15

beh_bel1 0.22904098 0.04669817 0.02917488 -0.01414449 0.30771632

beh_bel2 0.38654025 0.00000000 -0.04614670 0.07435894 0.32982539

beh_bel3 0.20881527 -0.02850171 -0.13598555 0.09901138 0.10322452

beh_bel4 0.42759300 0.03644583 0.19549537 -0.01246899 0.01919937

beh_bel5 0.09509579 -0.03611222 -0.04807849 -0.19696934 0.17673198

beh_bel6 0.32302677 -0.03102897 0.09110366 -0.12892622 0.19196719

beh_bel7 0.37925012 0.22486252 -0.02194361 -0.01574366 0.24453990

beh_bel8 0.69599726 0.23238037 0.28342437 0.24928933 0.29087339

beh_bel9 0.56523636 0.11320718 0.12662045 0.15717228 0.30813358

beh_bel10 0.63572837 0.10355086 0.19438631 0.09387284 0.25286245

beh_bel11 1.00000000 0.12762307 0.32114154 0.23688404 0.26064239

beh_bel12 0.12762307 1.00000000 0.47564810 0.39543193 0.18829284

beh_bel13 0.32114154 0.47564810 1.00000000 0.39203950 0.20009015

beh_bel14 0.23688404 0.39543193 0.39203950 1.00000000 0.03091672

beh_bel15 0.26064239 0.18829284 0.20009015 0.03091672 1.00000000

beh_bel16 0.38085545 0.49812586 0.55010508 0.49877838 0.27443662

beh_bel17 0.16252682 0.16336801 0.31098186 0.31436656 0.16891712

beh_bel18 0.47766141 0.37535856 0.50199406 0.41057308 0.34204887

beh_bel16 beh_bel17 beh_bel18

beh_bel1 0.08413153 0.099986791 0.08704700

beh_bel2 0.06989996 0.061679455 0.05746879

beh_bel3 -0.04003380 -0.026691676 -0.03857963

beh_bel4 0.11901609 0.119340164 0.04077083

beh_bel5 -0.17115493 -0.180149711 -0.15781016

beh_bel6 0.09851494 -0.224286154 0.12517798

beh_bel7 0.21231445 -0.006618992 0.16929059

beh_bel8 0.46976906 0.128351478 0.44944527

beh_bel9 0.34488763 0.104983938 0.31404093

beh_bel10 0.35191177 0.016145423 0.39113653

beh_bel11 0.38085545 0.162526819 0.47766141

beh_bel12 0.49812586 0.163368011 0.37535856

beh_bel13 0.55010508 0.310981864 0.50199406

beh_bel14 0.49877838 0.314366556 0.41057308

beh_bel15 0.27443662 0.168917120 0.34204887

beh_bel16 1.00000000 0.397288522 0.70599960

beh_bel17 0.39728852 1.000000000 0.46720635

beh_bel18 0.70599960 0.467206352 1.00000000

- Normative Beliefs

inj_pos_1 inj_pos_2 inj_pos_3 inj_pos_4 inj_pos_5

inj_pos_1 1.000000000 0.61531948 0.34748119 0.50211305 0.258676693

inj_pos_2 0.615319480 1.00000000 0.08579983 0.59616565 0.196672934

inj_pos_3 0.347481186 0.08579983 1.00000000 0.30668999 0.312378414

inj_pos_4 0.502113052 0.59616565 0.30668999 1.00000000 0.458109371

inj_pos_5 0.258676693 0.19667293 0.31237841 0.45810937 1.000000000

inj_pos_6 0.268290640 0.19376495 0.44955373 0.44173433 0.627030220

inj_pos_7 0.061174690 0.06182722 0.34503861 0.40180876 0.703372644

inj_pos_8 0.117884298 0.12762911 0.32662151 0.43977615 0.676513988

inj_neg_1 0.163998667 0.12859326 0.15046611 0.04411320 0.065400294

inj_neg_2 0.100060981 0.06766356 0.06237815 -0.07172400 -0.037879902

inj_neg_3 0.132586748 0.08058400 -0.05543963 -0.02220413 -0.010138559

desc_pos_1 0.205954185 0.17931686 -0.04460571 0.23773274 0.032732113

desc_pos_2 0.288229437 0.29004417 0.25993338 0.28645830 0.142541155

desc_pos_3 0.168099544 0.29097528 0.04854283 0.20494389 0.152611282

desc_pos_4 0.224318313 0.17484340 0.05379435 0.24020529 0.348510618

desc_pos_5 0.190032829 0.08630392 0.12551322 0.13344758 0.241777591

desc_pos_6 0.340425055 0.30864414 0.13608236 0.40754743 0.416620657

desc_pos_7 0.315997961 0.11071317 0.27170312 0.07640653 0.104199901

desc_neg_1 -0.166296849 -0.09297856 -0.24145270 -0.31067236 -0.093741662

desc_neg_2 -0.196553407 -0.16069468 -0.12487107 -0.23798167 -0.208184058

desc_neg_3 0.124432571 0.01836961 0.24210549 -0.02205206 0.113761356

desc_neg_4 0.156150029 0.14429218 -0.01803685 0.09793228 0.084607618

desc_neg_5 0.164570137 -0.03424306 0.08522571 0.06483765 0.032727262

desc_neg_6 -0.055433175 -0.22123169 0.06018887 0.02588799 0.001725453

desc_neg_7 -0.130092210 -0.20717800 -0.04398122 0.01955182 0.103805574

desc_neg_8 -0.007204686 -0.15310693 0.20181125 -0.11688685 0.102058257

inj_pos_6 inj_pos_7 inj_pos_8 inj_neg_1 inj_neg_2

inj_pos_1 0.26829064 0.061174690 0.117884298 0.16399867 0.10006098

inj_pos_2 0.19376495 0.061827220 0.127629112 0.12859326 0.06766356

inj_pos_3 0.44955373 0.345038613 0.326621506 0.15046611 0.06237815

inj_pos_4 0.44173433 0.401808759 0.439776153 0.04411320 -0.07172400

inj_pos_5 0.62703022 0.703372644 0.676513988 0.06540029 -0.03787990

inj_pos_6 1.00000000 0.684583515 0.631448744 0.10854887 -0.04203885

inj_pos_7 0.68458352 1.000000000 0.876893507 -0.01498274 -0.11794280

inj_pos_8 0.63144874 0.876893507 1.000000000 -0.07147657 -0.16449493

inj_neg_1 0.10854887 -0.014982744 -0.071476574 1.00000000 0.88004156

inj_neg_2 -0.04203885 -0.117942801 -0.164494926 0.88004156 1.00000000

inj_neg_3 -0.05722302 -0.043517843 -0.083723145 0.70300846 0.79285783

desc_pos_1 0.02253795 -0.069147667 -0.035040411 0.27881724 0.27429862

desc_pos_2 0.24006571 0.075921628 0.088479195 0.36590800 0.26646047

desc_pos_3 0.18182534 0.028730481 0.062243496 0.20165053 0.09652676

desc_pos_4 0.20504596 0.263441994 0.316211200 0.18570459 0.05778255

desc_pos_5 0.20355956 0.226073469 0.216577681 0.24333321 0.08072985

desc_pos_6 0.21166114 0.195874521 0.246161640 0.08511160 -0.03287697

desc_pos_7 0.15322409 0.112676780 0.215326250 0.20432538 0.20598219

desc_neg_1 -0.17732015 0.005770672 -0.048592456 -0.01582986 -0.05670053

desc_neg_2 -0.10771862 -0.053749809 -0.087335189 0.10239741 0.07900595

desc_neg_3 0.13305883 0.056818802 0.094091443 0.41805046 0.39223380

desc_neg_4 0.04092208 0.048313172 0.005991135 0.37187962 0.32979666

desc_neg_5 0.05388831 0.105822780 0.100237864 0.01409582 0.07258507

desc_neg_6 0.09164088 0.158085093 0.132302560 -0.04203701 0.02955740

desc_neg_7 0.15552150 0.232602463 0.245215011 -0.03411666 0.02586591

desc_neg_8 0.11271354 0.105921975 0.138417282 0.10611082 0.16863618

inj_neg_3 desc_pos_1 desc_pos_2 desc_pos_3 desc_pos_4

inj_pos_1 0.132586748 0.205954185 0.288229437 0.16809954 0.224318313

inj_pos_2 0.080584000 0.179316858 0.290044168 0.29097528 0.174843395

inj_pos_3 -0.055439629 -0.044605710 0.259933376 0.04854283 0.053794354

inj_pos_4 -0.022204128 0.237732740 0.286458299 0.20494389 0.240205293

inj_pos_5 -0.010138559 0.032732113 0.142541155 0.15261128 0.348510618

inj_pos_6 -0.057223019 0.022537947 0.240065711 0.18182534 0.205045963

inj_pos_7 -0.043517843 -0.069147667 0.075921628 0.02873048 0.263441994

inj_pos_8 -0.083723145 -0.035040411 0.088479195 0.06224350 0.316211200

inj_neg_1 0.703008461 0.278817244 0.365907998 0.20165053 0.185704595

inj_neg_2 0.792857831 0.274298621 0.266460467 0.09652676 0.057782548

inj_neg_3 1.000000000 0.245124615 0.212023971 -0.06838782 0.046337869

desc_pos_1 0.245124615 1.000000000 0.516386100 0.36153201 0.286770287

desc_pos_2 0.212023971 0.516386100 1.000000000 0.55655380 0.244569468

desc_pos_3 -0.068387817 0.361532010 0.556553801 1.00000000 0.214710510

desc_pos_4 0.046337869 0.286770287 0.244569468 0.21471051 1.000000000

desc_pos_5 0.097975845 0.297359882 0.176724548 0.11211352 0.755326771

desc_pos_6 -0.103492486 0.240378276 0.263130248 0.32450309 0.481904466

desc_pos_7 0.217188246 0.032524548 0.086415428 0.11278958 0.143293862

desc_neg_1 -0.067384458 -0.131016458 -0.230891911 -0.27357074 -0.058533450

desc_neg_2 -0.031985472 -0.056363705 -0.118320093 -0.28396507 -0.077745790

desc_neg_3 0.289842281 0.179351686 0.233194349 0.09101206 0.009701584

desc_neg_4 0.332418546 0.004690989 -0.004297065 -0.16101177 0.059313452

desc_neg_5 0.166448925 -0.056405884 -0.121207673 -0.18274743 -0.037300443

desc_neg_6 0.031134424 -0.117971261 -0.220936844 -0.30338286 -0.175515097

desc_neg_7 0.005642909 -0.051059158 -0.229752059 -0.13448869 -0.128083135

desc_neg_8 0.118694675 -0.134783058 0.018587296 -0.02142982 -0.058465731

desc_pos_5 desc_pos_6 desc_pos_7 desc_neg_1 desc_neg_2

inj_pos_1 0.190032829 0.34042505 0.31599796 -0.166296849 -0.19655341

inj_pos_2 0.086303921 0.30864414 0.11071317 -0.092978561 -0.16069468

inj_pos_3 0.125513217 0.13608236 0.27170312 -0.241452700 -0.12487107

inj_pos_4 0.133447584 0.40754743 0.07640653 -0.310672359 -0.23798167

inj_pos_5 0.241777591 0.41662066 0.10419990 -0.093741662 -0.20818406

inj_pos_6 0.203559564 0.21166114 0.15322409 -0.177320153 -0.10771862

inj_pos_7 0.226073469 0.19587452 0.11267678 0.005770672 -0.05374981

inj_pos_8 0.216577681 0.24616164 0.21532625 -0.048592456 -0.08733519

inj_neg_1 0.243333211 0.08511160 0.20432538 -0.015829857 0.10239741

inj_neg_2 0.080729851 -0.03287697 0.20598219 -0.056700527 0.07900595

inj_neg_3 0.097975845 -0.10349249 0.21718825 -0.067384458 -0.03198547

desc_pos_1 0.297359882 0.24037828 0.03252455 -0.131016458 -0.05636370

desc_pos_2 0.176724548 0.26313025 0.08641543 -0.230891911 -0.11832009

desc_pos_3 0.112113516 0.32450309 0.11278958 -0.273570744 -0.28396507

desc_pos_4 0.755326771 0.48190447 0.14329386 -0.058533450 -0.07774579

desc_pos_5 1.000000000 0.39044805 0.13424274 -0.017127949 -0.01310907

desc_pos_6 0.390448050 1.00000000 0.23862990 -0.211453383 -0.33727205

desc_pos_7 0.134242738 0.23862990 1.00000000 -0.228013304 -0.27035655

desc_neg_1 -0.017127949 -0.21145338 -0.22801330 1.000000000 0.47303097

desc_neg_2 -0.013109074 -0.33727205 -0.27035655 0.473030967 1.00000000

desc_neg_3 0.137992660 0.02425201 0.09629747 0.008162886 0.06243159

desc_neg_4 0.124179108 -0.01186603 -0.00369196 0.125793069 0.24909026

desc_neg_5 -0.007055974 -0.05439388 0.07929313 0.092753894 0.18926005

desc_neg_6 -0.113342521 -0.24500064 -0.02869247 0.182208399 0.28378868

desc_neg_7 -0.023467263 -0.21759017 -0.07196685 0.288771181 0.33768080

desc_neg_8 0.023453685 -0.06946684 0.18093425 0.092582956 0.12027457

desc_neg_3 desc_neg_4 desc_neg_5 desc_neg_6

inj_pos_1 0.124432571 0.156150029 0.164570137 -0.055433175

inj_pos_2 0.018369608 0.144292180 -0.034243062 -0.221231690

inj_pos_3 0.242105485 -0.018036847 0.085225708 0.060188867

inj_pos_4 -0.022052059 0.097932284 0.064837651 0.025887994

inj_pos_5 0.113761356 0.084607618 0.032727262 0.001725453

inj_pos_6 0.133058834 0.040922084 0.053888313 0.091640877

inj_pos_7 0.056818802 0.048313172 0.105822780 0.158085093

inj_pos_8 0.094091443 0.005991135 0.100237864 0.132302560

inj_neg_1 0.418050463 0.371879622 0.014095820 -0.042037012

inj_neg_2 0.392233800 0.329796655 0.072585072 0.029557403

inj_neg_3 0.289842281 0.332418546 0.166448925 0.031134424

desc_pos_1 0.179351686 0.004690989 -0.056405884 -0.117971261

desc_pos_2 0.233194349 -0.004297065 -0.121207673 -0.220936844

desc_pos_3 0.091012065 -0.161011769 -0.182747425 -0.303382863

desc_pos_4 0.009701584 0.059313452 -0.037300443 -0.175515097

desc_pos_5 0.137992660 0.124179108 -0.007055974 -0.113342521

desc_pos_6 0.024252009 -0.011866032 -0.054393880 -0.245000641

desc_pos_7 0.096297473 -0.003691960 0.079293125 -0.028692470

desc_neg_1 0.008162886 0.125793069 0.092753894 0.182208399

desc_neg_2 0.062431593 0.249090265 0.189260052 0.283788682

desc_neg_3 1.000000000 0.499417368 0.287477182 0.118083470

desc_neg_4 0.499417368 1.000000000 0.615466017 0.474058704

desc_neg_5 0.287477182 0.615466017 1.000000000 0.717947582

desc_neg_6 0.118083470 0.474058704 0.717947582 1.000000000

desc_neg_7 0.153139214 0.129650040 0.325869163 0.521897220

desc_neg_8 0.350596440 0.039332641 0.076513296 0.110161053

desc_neg_7 desc_neg_8

inj_pos_1 -0.130092210 -0.007204686

inj_pos_2 -0.207177997 -0.153106928

inj_pos_3 -0.043981217 0.201811253

inj_pos_4 0.019551820 -0.116886849

inj_pos_5 0.103805574 0.102058257

inj_pos_6 0.155521501 0.112713544

inj_pos_7 0.232602463 0.105921975

inj_pos_8 0.245215011 0.138417282

inj_neg_1 -0.034116661 0.106110816

inj_neg_2 0.025865909 0.168636179

inj_neg_3 0.005642909 0.118694675

desc_pos_1 -0.051059158 -0.134783058

desc_pos_2 -0.229752059 0.018587296

desc_pos_3 -0.134488691 -0.021429821

desc_pos_4 -0.128083135 -0.058465731

desc_pos_5 -0.023467263 0.023453685

desc_pos_6 -0.217590169 -0.069466844

desc_pos_7 -0.071966847 0.180934250

desc_neg_1 0.288771181 0.092582956

desc_neg_2 0.337680800 0.120274572

desc_neg_3 0.153139214 0.350596440

desc_neg_4 0.129650040 0.039332641

desc_neg_5 0.325869163 0.076513296

desc_neg_6 0.521897220 0.110161053

desc_neg_7 1.000000000 0.382079948

desc_neg_8 0.382079948 1.000000000

- Control Beliefs

fac1 fac2 fac3 fac4 fac5

fac1 1.00000000 0.59797536 0.20215139 0.38227354 0.31669897

fac2 0.59797536 1.00000000 0.24839211 0.58026615 0.38223516

fac3 0.20215139 0.24839211 1.00000000 0.40671759 0.16898761

fac4 0.38227354 0.58026615 0.40671759 1.00000000 0.40066895

fac5 0.31669897 0.38223516 0.16898761 0.40066895 1.00000000

fac6 0.25022903 0.40454505 0.17797403 0.40682578 0.33177892

fac7 0.43310848 0.50431029 0.31013262 0.43254383 0.34384371

bar1 0.08005553 -0.06538211 -0.09149164 -0.09072311 -0.04648731

bar2 -0.07895995 -0.12366386 -0.08536605 -0.10169734 -0.10724649

bar3 -0.20422951 -0.15885913 -0.06068563 -0.10403433 -0.08954347

bar4 -0.10517136 -0.19927511 -0.24475275 -0.19750769 -0.24319326

bar5 -0.27739422 -0.22313193 -0.24246112 -0.12250027 -0.17786309

bar6 -0.19621470 -0.39862920 -0.06189025 -0.38931679 -0.25579020

fac6 fac7 bar1 bar2 bar3

fac1 0.25022903 0.43310848 0.08005553 -0.07895995 -0.20422951

fac2 0.40454505 0.50431029 -0.06538211 -0.12366386 -0.15885913

fac3 0.17797403 0.31013262 -0.09149164 -0.08536605 -0.06068563

fac4 0.40682578 0.43254383 -0.09072311 -0.10169734 -0.10403433

fac5 0.33177892 0.34384371 -0.04648731 -0.10724649 -0.08954347

fac6 1.00000000 0.31165990 -0.07573504 -0.08942661 -0.06434513

fac7 0.31165990 1.00000000 -0.07991544 -0.12396513 -0.24560080

bar1 -0.07573504 -0.07991544 1.00000000 0.23477637 0.23227853

bar2 -0.08942661 -0.12396513 0.23477637 1.00000000 0.89762761

bar3 -0.06434513 -0.24560080 0.23227853 0.89762761 1.00000000

bar4 -0.21330763 -0.22001920 0.24703981 0.27593645 0.31159007

bar5 -0.16308393 -0.17315548 0.14727265 0.38975513 0.42458114

bar6 -0.26010427 -0.36540086 0.14657604 0.28576493 0.36026183

bar4 bar5 bar6

fac1 -0.1051714 -0.2773942 -0.19621470

fac2 -0.1992751 -0.2231319 -0.39862920

fac3 -0.2447528 -0.2424611 -0.06189025

fac4 -0.1975077 -0.1225003 -0.38931679

fac5 -0.2431933 -0.1778631 -0.25579020

fac6 -0.2133076 -0.1630839 -0.26010427

fac7 -0.2200192 -0.1731555 -0.36540086

bar1 0.2470398 0.1472726 0.14657604

bar2 0.2759365 0.3897551 0.28576493

bar3 0.3115901 0.4245811 0.36026183

bar4 1.0000000 0.5959076 0.50308820

bar5 0.5959076 1.0000000 0.51503957

bar6 0.5030882 0.5150396 1.00000000

- Habit Beliefs

exp1 exp2 exp3 exp4 auto1

exp1 1.000000000 0.45316809 0.14315464 0.23948184 -0.002948333

exp2 0.453168089 1.00000000 0.07948175 0.33830506 -0.035309787

exp3 0.143154635 0.07948175 1.00000000 0.60598169 0.133753184

exp4 0.239481835 0.33830506 0.60598169 1.00000000 0.096958186

auto1 -0.002948333 -0.03530979 0.13375318 0.09695819 1.000000000

auto2 0.057922134 0.07035743 0.35849358 0.25468919 0.493062277

auto3 0.179542082 0.16754845 0.39890063 0.28951694 0.459832896

auto2 auto3

exp1 0.05792213 0.1795421

exp2 0.07035743 0.1675484

exp3 0.35849358 0.3989006

exp4 0.25468919 0.2895169

auto1 0.49306228 0.4598329

auto2 1.00000000 0.8048035

auto3 0.80480352 1.0000000

- Moral Norm Beliefs

resp1 resp2 val1 val2 val3

resp1 1.00000000 0.16045605 -0.1245377 0.05169327 0.1403929

resp2 0.16045605 1.00000000 0.4755495 -0.01676625 0.5614638

val1 -0.12453773 0.47554946 1.0000000 0.10623381 0.4438833

val2 0.05169327 -0.01676625 0.1062338 1.00000000 0.1805532

val3 0.14039290 0.56146380 0.4438833 0.18055319 1.0000000

Reducing car use

- Behavioural Beliefs

beh_bel1 beh_bel2 beh_bel3 beh_bel4 beh_bel5

beh_bel1 1.00000000 0.75859845 0.82434924 0.10607959 0.29995884

beh_bel2 0.75859845 1.00000000 0.77804603 0.11394412 0.38856690

beh_bel3 0.82434924 0.77804603 1.00000000 0.02109491 0.31196703

beh_bel4 0.10607959 0.11394412 0.02109491 1.00000000 0.41552198

beh_bel5 0.29995884 0.38856690 0.31196703 0.41552198 1.00000000

beh_bel6 0.41669852 0.36197096 0.41230651 0.39905527 0.68492516

beh_bel7 0.34159802 0.26186485 0.33018127 0.38052871 0.35413135

beh_bel8 0.21280685 0.17852483 0.17775788 0.38449816 0.18374299

beh_bel9 -0.15980341 -0.21234388 -0.09003580 0.19578261 0.08208266

beh_bel10 0.08933853 0.06571400 0.03399513 0.25646211 0.10492173

beh_bel11 0.21926474 0.22402122 0.24260815 0.13737435 0.11176417

beh_bel12 0.35112554 0.30591689 0.31235104 0.28076745 0.19057177

beh_bel13 0.12650363 0.07643712 0.10891105 0.23295201 -0.06271962

beh_bel14 0.07993816 0.06172132 0.06072405 0.26839842 0.02636594

beh_bel6 beh_bel7 beh_bel8 beh_bel9 beh_bel10 beh_bel11

beh_bel1 0.41669852 0.3415980 0.2128068 -0.15980341 0.08933853 0.2192647

beh_bel2 0.36197096 0.2618649 0.1785248 -0.21234388 0.06571400 0.2240212

beh_bel3 0.41230651 0.3301813 0.1777579 -0.09003580 0.03399513 0.2426081

beh_bel4 0.39905527 0.3805287 0.3844982 0.19578261 0.25646211 0.1373744

beh_bel5 0.68492516 0.3541313 0.1837430 0.08208266 0.10492173 0.1117642

beh_bel6 1.00000000 0.3474713 0.2373128 0.09759081 0.18209133 0.1136992

beh_bel7 0.34747126 1.0000000 0.6046528 0.21747513 0.35013752 0.3106774

beh_bel8 0.23731282 0.6046528 1.0000000 0.33492316 0.48013821 0.3110846

beh_bel9 0.09759081 0.2174751 0.3349232 1.00000000 0.51424765 0.1418050

beh_bel10 0.18209133 0.3501375 0.4801382 0.51424765 1.00000000 0.4997852

beh_bel11 0.11369920 0.3106774 0.3110846 0.14180497 0.49978518 1.0000000

beh_bel12 0.26739619 0.5991616 0.5935553 0.20656778 0.49153216 0.5048743

beh_bel13 0.14280110 0.3788168 0.4638259 0.44600137 0.55450483 0.3814443

beh_bel14 0.11772533 0.1671093 0.3650986 0.53336577 0.61402117 0.4276457

beh_bel12 beh_bel13 beh_bel14

beh_bel1 0.3511255 0.12650363 0.07993816

beh_bel2 0.3059169 0.07643712 0.06172132

beh_bel3 0.3123510 0.10891105 0.06072405

beh_bel4 0.2807675 0.23295201 0.26839842

beh_bel5 0.1905718 -0.06271962 0.02636594

beh_bel6 0.2673962 0.14280110 0.11772533

beh_bel7 0.5991616 0.37881679 0.16710930

beh_bel8 0.5935553 0.46382586 0.36509858

beh_bel9 0.2065678 0.44600137 0.53336577

beh_bel10 0.4915322 0.55450483 0.61402117

beh_bel11 0.5048743 0.38144430 0.42764571

beh_bel12 1.0000000 0.45216956 0.40469498

beh_bel13 0.4521696 1.00000000 0.57769139

beh_bel14 0.4046950 0.57769139 1.00000000

- Normative Beliefs

inj_pos_1 inj_pos_2 inj_pos_3 inj_neg_1

inj_pos_1 1.000000000 0.028661597 -0.0785240247 0.068653301

inj_pos_2 0.028661597 1.000000000 0.4251178537 -0.148316384

inj_pos_3 -0.078524025 0.425117854 1.0000000000 -0.107818670

inj_neg_1 0.068653301 -0.148316384 -0.1078186699 1.000000000

inj_neg_2 0.023192678 -0.016448914 0.1939944031 -0.189696869

inj_neg_3 0.162152986 0.001446241 0.0794680666 0.073747263

desc_pos_1 0.283771025 0.059010774 0.0604509157 0.012746892

desc_pos_2 0.276156863 0.161135346 0.1606403798 0.127025838

desc_pos_3 0.309568898 0.154035736 0.2313159860 0.033065942

desc_pos_4 -0.006283718 0.047596797 0.1324840329 -0.007225916

desc_pos_5 -0.014330295 0.046680128 0.2203283478 0.097226216

desc_neg_1 -0.033790575 -0.017924572 -0.0002211143 0.080412874

desc_neg_2 0.060726562 -0.229368292 -0.0062527273 0.043471621

desc_neg_3 -0.048051252 -0.056667911 0.1059999684 -0.004711768

inj_neg_2 inj_neg_3 desc_pos_1 desc_pos_2 desc_pos_3

inj_pos_1 0.023192678 0.162152986 0.28377102 0.276156863 0.30956890

inj_pos_2 -0.016448914 0.001446241 0.05901077 0.161135346 0.15403574

inj_pos_3 0.193994403 0.079468067 0.06045092 0.160640380 0.23131599

inj_neg_1 -0.189696869 0.073747263 0.01274689 0.127025838 0.03306594

inj_neg_2 1.000000000 0.400704837 -0.11674037 -0.032493886 -0.06033445

inj_neg_3 0.400704837 1.000000000 -0.04588659 0.092325277 0.08989084

desc_pos_1 -0.116740367 -0.045886591 1.00000000 0.216509038 0.18483773

desc_pos_2 -0.032493886 0.092325277 0.21650904 1.000000000 0.82161327

desc_pos_3 -0.060334448 0.089890840 0.18483773 0.821613272 1.00000000

desc_pos_4 -0.070963292 0.028975437 -0.06160182 0.180958301 0.21017859

desc_pos_5 -0.001191807 0.091009846 0.26915773 0.162658146 0.15507460

desc_neg_1 -0.034388710 -0.016624186 -0.19156347 0.008150433 0.04489992

desc_neg_2 0.234716305 0.134995504 -0.35035042 -0.100917894 -0.11641557

desc_neg_3 0.193430599 0.132686512 -0.11710250 -0.153657612 -0.22004974

desc_pos_4 desc_pos_5 desc_neg_1 desc_neg_2

inj_pos_1 -0.006283718 -0.014330295 -0.0337905747 0.060726562

inj_pos_2 0.047596797 0.046680128 -0.0179245717 -0.229368292

inj_pos_3 0.132484033 0.220328348 -0.0002211143 -0.006252727

inj_neg_1 -0.007225916 0.097226216 0.0804128741 0.043471621

inj_neg_2 -0.070963292 -0.001191807 -0.0343887098 0.234716305

inj_neg_3 0.028975437 0.091009846 -0.0166241864 0.134995504

desc_pos_1 -0.061601816 0.269157728 -0.1915634683 -0.350350421

desc_pos_2 0.180958301 0.162658146 0.0081504331 -0.100917894

desc_pos_3 0.210178586 0.155074602 0.0448999173 -0.116415570

desc_pos_4 1.000000000 0.355354595 0.4196711313 0.176763970

desc_pos_5 0.355354595 1.000000000 0.0728172679 -0.093983640

desc_neg_1 0.419671131 0.072817268 1.0000000000 0.190215199

desc_neg_2 0.176763970 -0.093983640 0.1902151987 1.000000000

desc_neg_3 -0.017407222 -0.069525032 -0.1440648368 0.578024390

desc_neg_3

inj_pos_1 -0.048051252

inj_pos_2 -0.056667911

inj_pos_3 0.105999968

inj_neg_1 -0.004711768

inj_neg_2 0.193430599

inj_neg_3 0.132686512

desc_pos_1 -0.117102495

desc_pos_2 -0.153657612

desc_pos_3 -0.220049739

desc_pos_4 -0.017407222

desc_pos_5 -0.069525032

desc_neg_1 -0.144064837

desc_neg_2 0.578024390

desc_neg_3 1.000000000

- Control Beliefs

fac1 fac2 bar1 bar2 bar3 bar4

fac1 1.00000000 0.69694292 -0.41078726 -0.4523995 -0.1835061 0.04817869

fac2 0.69694292 1.00000000 -0.09947305 -0.1936797 -0.1718205 -0.20359261

bar1 -0.41078726 -0.09947305 1.00000000 0.8574172 0.2528145 0.13100561

bar2 -0.45239953 -0.19367966 0.85741719 1.0000000 0.3268320 0.12784897

bar3 -0.18350613 -0.17182049 0.25281454 0.3268320 1.0000000 0.37041564

bar4 0.04817869 -0.20359261 0.13100561 0.1278490 0.3704156 1.00000000

- Habit Beliefs

exp1 exp2 exp3 exp4 exp5

exp1 1.00000000 0.057998585 0.647328278 0.12884884 0.50629677

exp2 0.05799858 1.000000000 0.004124393 0.77864513 -0.11187004

exp3 0.64732828 0.004124393 1.000000000 0.04711471 0.39654951

exp4 0.12884884 0.778645131 0.047114710 1.00000000 -0.07952203

exp5 0.50629677 -0.111870043 0.396549509 -0.07952203 1.00000000

auto1 0.51484110 -0.114918919 0.231768776 -0.11500476 0.31552859

auto2 0.42593676 -0.219736762 0.400508970 -0.16713586 0.37474219

auto3 0.46809834 0.050415682 0.388438535 0.03191776 0.22198144

auto4 0.38696907 0.047675699 0.408643400 0.06692434 0.37600117

auto1 auto2 auto3 auto4

exp1 0.5148411 0.4259368 0.46809834 0.38696907

exp2 -0.1149189 -0.2197368 0.05041568 0.04767570

exp3 0.2317688 0.4005090 0.38843854 0.40864340

exp4 -0.1150048 -0.1671359 0.03191776 0.06692434

exp5 0.3155286 0.3747422 0.22198144 0.37600117

auto1 1.0000000 0.3920445 0.18741263 0.26825729

auto2 0.3920445 1.0000000 0.30798205 0.33011250

auto3 0.1874126 0.3079820 1.00000000 0.42953020

auto4 0.2682573 0.3301125 0.42953020 1.00000000

- Moral Norm Beliefs

resp1 resp2 resp3 val1 val2

resp1 1.0000000 0.6320530 0.6515318 0.7498227 0.5042403

resp2 0.6320530 1.0000000 0.6453843 0.5372075 0.3990396

resp3 0.6515318 0.6453843 1.0000000 0.6864016 0.4691420

val1 0.7498227 0.5372075 0.6864016 1.0000000 0.5711523

val2 0.5042403 0.3990396 0.4691420 0.5711523 1.0000000

Reducing flights

- Behavioural Beliefs

beh_bel1 beh_bel2 beh_bel3 beh_bel4 beh_bel5

beh_bel1 1.00000000 0.724265427 0.846296778 0.193855177 0.59738499

beh_bel2 0.72426543 1.000000000 0.631256858 0.304196974 0.54621604

beh_bel3 0.84629678 0.631256858 1.000000000 0.193154557 0.62540155

beh_bel4 0.19385518 0.304196974 0.193154557 1.000000000 0.24810224

beh_bel5 0.59738499 0.546216044 0.625401552 0.248102236 1.00000000

beh_bel6 0.13820111 0.128101241 0.228591350 0.351974747 0.28452951

beh_bel7 0.32756339 0.274899687 0.369936930 0.220166848 0.42735468

beh_bel8 0.40390167 0.334035571 0.384069923 0.160473968 0.22732735

beh_bel9 0.05326289 -0.029205101 0.050763672 0.230202469 0.07287137

beh_bel10 0.12702622 0.030838353 0.182328658 0.332993081 0.21430571

beh_bel11 -0.01938726 0.043474678 -0.006784723 0.183416479 0.11487248

beh_bel12 0.22476370 0.112173487 0.192235126 -0.003884601 0.21095403

beh_bel13 0.01746363 0.047932432 -0.009768451 0.370533410 0.09559174

beh_bel14 0.13348694 0.118118919 0.138823137 0.245490653 0.16831561

beh_bel15 0.08109932 -0.004154674 0.069663946 0.145251964 0.14349968

beh_bel16 0.21783396 0.150704037 0.237114615 0.210159127 0.21709393

beh_bel6 beh_bel7 beh_bel8 beh_bel9 beh_bel10

beh_bel1 0.13820111 0.3275634 0.40390167 0.05326289 0.12702622

beh_bel2 0.12810124 0.2748997 0.33403557 -0.02920510 0.03083835

beh_bel3 0.22859135 0.3699369 0.38406992 0.05076367 0.18232866

beh_bel4 0.35197475 0.2201668 0.16047397 0.23020247 0.33299308

beh_bel5 0.28452951 0.4273547 0.22732735 0.07287137 0.21430571

beh_bel6 1.00000000 0.4427510 0.18778924 0.07832104 0.34135969

beh_bel7 0.44275101 1.0000000 0.19864754 0.23540722 0.58290791

beh_bel8 0.18778924 0.1986475 1.00000000 0.17410105 0.30401700

beh_bel9 0.07832104 0.2354072 0.17410105 1.00000000 0.61555218

beh_bel10 0.34135969 0.5829079 0.30401700 0.61555218 1.00000000

beh_bel11 0.15453365 0.2323980 0.12228888 0.26373237 0.34659672

beh_bel12 0.19340964 0.3163225 0.03343512 0.31627878 0.30700698

beh_bel13 0.16335619 0.1656358 0.11196402 0.37941117 0.36237573

beh_bel14 0.28604118 0.4671944 0.18017830 0.24109859 0.48623466

beh_bel15 0.31883744 0.4767287 0.18684786 0.37291700 0.44583785

beh_bel16 0.21111954 0.4614867 0.10406965 0.32000974 0.39773715

beh_bel11 beh_bel12 beh_bel13 beh_bel14 beh_bel15

beh_bel1 -0.019387263 0.224763704 0.017463632 0.1334869 0.081099320

beh_bel2 0.043474678 0.112173487 0.047932432 0.1181189 -0.004154674

beh_bel3 -0.006784723 0.192235126 -0.009768451 0.1388231 0.069663946

beh_bel4 0.183416479 -0.003884601 0.370533410 0.2454907 0.145251964

beh_bel5 0.114872481 0.210954031 0.095591745 0.1683156 0.143499681

beh_bel6 0.154533650 0.193409637 0.163356192 0.2860412 0.318837436

beh_bel7 0.232398033 0.316322539 0.165635756 0.4671944 0.476728749

beh_bel8 0.122288881 0.033435121 0.111964015 0.1801783 0.186847855

beh_bel9 0.263732366 0.316278780 0.379411169 0.2410986 0.372917001

beh_bel10 0.346596721 0.307006985 0.362375729 0.4862347 0.445837847

beh_bel11 1.000000000 0.258541582 0.113447135 0.5403948 0.437859179

beh_bel12 0.258541582 1.000000000 0.396927577 0.4637950 0.509575301

beh_bel13 0.113447135 0.396927577 1.000000000 0.3564809 0.393943838

beh_bel14 0.540394766 0.463794953 0.356480917 1.0000000 0.683358146

beh_bel15 0.437859179 0.509575301 0.393943838 0.6833581 1.000000000

beh_bel16 0.506071965 0.479199953 0.249070320 0.7299610 0.618996188

beh_bel16

beh_bel1 0.2178340

beh_bel2 0.1507040

beh_bel3 0.2371146

beh_bel4 0.2101591

beh_bel5 0.2170939

beh_bel6 0.2111195

beh_bel7 0.4614867

beh_bel8 0.1040696

beh_bel9 0.3200097

beh_bel10 0.3977372

beh_bel11 0.5060720

beh_bel12 0.4792000

beh_bel13 0.2490703

beh_bel14 0.7299610

beh_bel15 0.6189962

beh_bel16 1.0000000

- Normative Beliefs

inj_pos_1 inj_pos_2 inj_pos_3 inj_pos_4 inj_pos_5

inj_pos_1 1.000000000 0.92892177 0.20774454 0.285778906 0.43867204

inj_pos_2 0.928921766 1.00000000 0.22848782 0.315620815 0.48353590

inj_pos_3 0.207744543 0.22848782 1.00000000 0.852435976 0.70656121

inj_pos_4 0.285778906 0.31562082 0.85243598 1.000000000 0.73961282

inj_pos_5 0.438672035 0.48353590 0.70656121 0.739612817 1.00000000

inj_neg_1 -0.256982503 -0.26962893 0.31480875 0.167553511 0.20452419

inj_neg_2 0.131860280 0.05503435 -0.03914267 0.000542368 0.15291541

inj_neg_3 0.102031729 0.04126849 0.05857137 -0.034546593 0.01302910

desc_pos_1 0.024621787 0.08445666 0.37510973 0.397823090 0.37420512

desc_pos_2 0.233516422 0.24472072 -0.04807409 0.001242324 0.04957657

desc_pos_3 0.295001047 0.24739373 -0.12475014 -0.106242955 0.00672159

desc_pos_4 0.244801238 0.23799564 0.12420311 0.063062069 0.07920136

desc_neg_1 -0.009641634 -0.02084386 0.02452558 0.085862025 0.05047934

desc_neg_2 -0.123098637 -0.13325599 0.03749497 -0.025074429 0.03176948

desc_neg_3 -0.183942910 -0.17842516 -0.03385721 -0.069281918 -0.01310553

desc_neg_4 -0.178337515 -0.16197570 -0.10995849 -0.095168278 -0.03523570

inj_neg_1 inj_neg_2 inj_neg_3 desc_pos_1 desc_pos_2

inj_pos_1 -0.25698250 0.131860280 0.102031729 0.02462179 0.233516422

inj_pos_2 -0.26962893 0.055034349 0.041268488 0.08445666 0.244720718

inj_pos_3 0.31480875 -0.039142667 0.058571368 0.37510973 -0.048074091

inj_pos_4 0.16755351 0.000542368 -0.034546593 0.39782309 0.001242324

inj_pos_5 0.20452419 0.152915408 0.013029100 0.37420512 0.049576565

inj_neg_1 1.00000000 0.333214562 0.199329458 0.29333492 -0.252944891

inj_neg_2 0.33321456 1.000000000 0.483798705 0.09253645 -0.014654834

inj_neg_3 0.19932946 0.483798705 1.000000000 0.15818961 0.078006328

desc_pos_1 0.29333492 0.092536451 0.158189614 1.00000000 -0.117376649

desc_pos_2 -0.25294489 -0.014654834 0.078006328 -0.11737665 1.000000000

desc_pos_3 -0.16600925 0.071397209 0.214549045 -0.04821611 0.747178097

desc_pos_4 -0.17680669 0.007744301 0.147801821 0.13765670 0.026096425

desc_neg_1 0.08795210 -0.066000254 -0.076453770 0.25086204 -0.004300731

desc_neg_2 0.16795498 -0.040367917 0.003293536 0.08776040 -0.009739986

desc_neg_3 0.18251169 -0.031567540 0.012940975 0.06560003 0.004315913

desc_neg_4 0.07897122 -0.071830085 -0.010109681 -0.03107698 -0.106006980

desc_pos_3 desc_pos_4 desc_neg_1 desc_neg_2

inj_pos_1 0.295001047 0.244801238 -0.009641634 -0.123098637

inj_pos_2 0.247393730 0.237995643 -0.020843861 -0.133255992

inj_pos_3 -0.124750140 0.124203115 0.024525583 0.037494972

inj_pos_4 -0.106242955 0.063062069 0.085862025 -0.025074429

inj_pos_5 0.006721590 0.079201364 0.050479339 0.031769475

inj_neg_1 -0.166009249 -0.176806693 0.087952105 0.167954982

inj_neg_2 0.071397209 0.007744301 -0.066000254 -0.040367917

inj_neg_3 0.214549045 0.147801821 -0.076453770 0.003293536

desc_pos_1 -0.048216107 0.137656696 0.250862035 0.087760395

desc_pos_2 0.747178097 0.026096425 -0.004300731 -0.009739986

desc_pos_3 1.000000000 0.231638002 0.006181007 0.053734162

desc_pos_4 0.231638002 1.000000000 -0.149157166 -0.141248917

desc_neg_1 0.006181007 -0.149157166 1.000000000 0.666810726

desc_neg_2 0.053734162 -0.141248917 0.666810726 1.000000000

desc_neg_3 0.052579885 -0.189574489 0.652439813 0.959259738

desc_neg_4 -0.055835821 -0.213408826 0.569826026 0.729121965

desc_neg_3 desc_neg_4

inj_pos_1 -0.183942910 -0.17833751

inj_pos_2 -0.178425159 -0.16197570

inj_pos_3 -0.033857212 -0.10995849

inj_pos_4 -0.069281918 -0.09516828

inj_pos_5 -0.013105529 -0.03523570

inj_neg_1 0.182511689 0.07897122

inj_neg_2 -0.031567540 -0.07183008

inj_neg_3 0.012940975 -0.01010968

desc_pos_1 0.065600030 -0.03107698

desc_pos_2 0.004315913 -0.10600698

desc_pos_3 0.052579885 -0.05583582

desc_pos_4 -0.189574489 -0.21340883

desc_neg_1 0.652439813 0.56982603

desc_neg_2 0.959259738 0.72912197

desc_neg_3 1.000000000 0.80021772

desc_neg_4 0.800217719 1.00000000

- Control Beliefs

fac1 fac2 fac3 fac4 bar1 bar2

fac1 1.00000000 0.81268070 0.25190959 0.2344225 -0.1146243 -0.11376144

fac2 0.81268070 1.00000000 0.19538001 0.2703043 -0.2141275 -0.21412777

fac3 0.25190959 0.19538001 1.00000000 0.2743824 0.1164825 0.07977902

fac4 0.23442251 0.27030428 0.27438235 1.0000000 -0.2009935 -0.16828185

bar1 -0.11462431 -0.21412749 0.11648248 -0.2009935 1.0000000 0.81202829

bar2 -0.11376144 -0.21412777 0.07977902 -0.1682819 0.8120283 1.00000000

bar3 -0.11639483 -0.21211207 -0.01586391 -0.4346018 0.2929222 0.20750421

bar4 -0.05979962 -0.08766198 -0.05124866 -0.1412130 0.1384188 0.02588104

bar3 bar4

fac1 -0.11639483 -0.05979962

fac2 -0.21211207 -0.08766198

fac3 -0.01586391 -0.05124866

fac4 -0.43460175 -0.14121305

bar1 0.29292223 0.13841877

bar2 0.20750421 0.02588104

bar3 1.00000000 0.14742779

bar4 0.14742779 1.00000000

- Habit Beliefs

exp1 exp2 auto1 auto2 auto3

exp1 1.0000000 0.6624567 0.4503237 0.2923329 0.3655708

exp2 0.6624567 1.0000000 0.1955337 0.1355963 0.3628423

auto1 0.4503237 0.1955337 1.0000000 0.3688943 0.2945409

auto2 0.2923329 0.1355963 0.3688943 1.0000000 0.3562101

auto3 0.3655708 0.3628423 0.2945409 0.3562101 1.0000000

- Moral Norm Beliefs

resp1 resp2 resp3 val1 val2 val3

resp1 1.00000000 0.021985048 0.45695633 0.611106759 0.35767419 0.53708123

resp2 0.02198505 1.000000000 0.01705307 0.008457793 0.06342957 0.07179257

resp3 0.45695633 0.017053065 1.00000000 0.401342497 0.25623085 0.23376644

val1 0.61110676 0.008457793 0.40134250 1.000000000 0.35195852 0.68107598

val2 0.35767419 0.063429568 0.25623085 0.351958521 1.00000000 0.35561194

val3 0.53708123 0.071792568 0.23376644 0.681075983 0.35561194 1.00000000

val4 0.52826729 0.078164773 0.56089546 0.301798125 0.32152409 0.25928651

val4

resp1 0.52826729

resp2 0.07816477

resp3 0.56089546

val1 0.30179812

val2 0.32152409

val3 0.25928651

val4 1.00000000
