# Supplementary material for: On the road to sustainability: Applying an extended Theory of Planned Behaviour model to energy-saving transportation practices
Source: PLoS One. 2025 Jun 3;20(6):e0325196. doi: 10.1371/journal.pone.0325196 (PMC12132967; doi:10.1371/journal.pone.0325196)
Supplement: S4 File — (DOCX) [file pone.0325196.s004.docx]

**S4 File:** Factor Analyses

Factor analyses of beliefs underlying use of sustainable transportation

| **Belief** | **Factor 1** | **Factor 2** | **Factor 3** |
| --- | --- | --- | --- |
| **Behavioural beliefs** |  |  |  |
| Choosing sustainable transportation would… | Advantages | Disadvantages |  |
| …help the environment | 0.85 |  |  |
| …reduce my carbon footprint | 0.86 |  |  |
| …lead to less air pollution | 0.67 |  |  |
| …lower my transportation costs | 0.43 |  |  |
| …improve my mental health | 0.57 |  |  |
| …give me a sense of intrinsic reward | 0.74 |  |  |
| …make me happy* |  |  |  |
| …make me feel eco-friendly | 1.02 |  |  |
| …make me feel morally good | 0.90 |  |  |
| …be inconvenient |  | 0.79 |  |
| …be expensive |  | 0.54 |  |
| …be unreliable |  | 0.83 |  |
| …be inaccessible |  | 0.80 |  |
| …be time consuming |  | 0.90 |  |
| …make me annoyed |  | 0.85 |  |
| …make me worried |  | 0.75 |  |
| Factor correlations*** | 0.67 | 0.67 |  |
| Alpha (α) | 0.92 | 0.90 |  |
|  |  |  |  |
| **Normative beliefs** |  |  |  |
| The following individuals or groups think I should choose sustainable transportation on a regular basis: | Young approvers | Professional approvers | Disapprovers |
| Environmentalists***** | 0.33 |  |  |
| Government representatives |  | 0.65 |  |
| General public* |  |  |  |
| Young people | 0.91 |  |  |
| Students | 0.89 |  |  |
| Commuters |  | 0.57 |  |
| Businessmen |  | 0.93 |  |
| The following people or groups of people think I should NOT choose sustainable transportation on a regular basis: |  |  |  |
| Workers |  |  | 0.62 |
| The motor industry |  |  | 0.81 |
| Non-environmentalists |  |  | 0.76 |
| The following people or groups of people are most likely to use sustainable transportation on a regular basis: |  |  |  |
| Young people | 0.65 |  |  |
| Climate activists**** |  |  |  |
| People concerned about the environment**** |  |  |  |
| The following people or groups of people are least likely to use sustainable transportation on a regular basis: |  |  |  |
| Elderly**** |  |  |  |
| Wealthy****** |  |  | 0.38 |
| People who live in isolated areas**** |  |  |  |
| Factor correlations*** | 0.02, -0.53 | 0.02, 0.34 | -0.53, 0.34 |
| Alpha (α) | 0.86 | 0.77 | 0.81 |
|  |  |  |  |
| **Control beliefs** |  |  |  |
| The following factors or circumstances would make it easier for me to choose sustainable transportation: | Facilitators | Barriers |  |
| Increased availability | 0.89 |  |  |
| Increased efficiency | 0.91 |  |  |
| Reduced cost | 0.78 |  |  |
| Increased accessibility | 0.86 |  |  |
| The following factors or circumstances would make it harder for me to choose sustainable transportation: |  |  |  |
| Timings |  | 0.66 |  |
| Unreliability |  | 0.75 |  |
| Inaccessibility |  | 0.71 |  |
| Inconvenience |  | 0.72 |  |
| Price |  | 0.48 |  |
| Factor correlations*** | -0.18 | -0.18 |  |
| Alpha (α) | 0.92 | 0.80 |  |
|  |  |  |  |
| **Habit beliefs** |  |  |  |
| Thinking about your behaviour over the last few years, how much do you agree with the following statements: | Experience |  |  |
| I tried sustainable transportation and it was a pleasant experience | 0.85 |  |  |
| I tried sustainable transportation when it was practical | 0.86 |  |  |
| I tried sustainable transportation but it was difficult** |  |  |  |
| I have not tried sustainable transportation because I pick the cheapest and most convenient option | 0.52 |  |  |
| I found myself automatically choosing sustainable transportation when... |  |  |  |
| …I was travelling long distances | 0.33 |  |  |
| …it was most convenient | 0.62 |  |  |
| …I was going to work | 0.57 |  |  |
| Alpha (α) | 0.79 |  |  |
|  |  |  |  |
| **Moral beliefs** |  |  |  |
| Thinking about individual responsibility to choose sustainable transportation, how much do you agree with the following statements: | Responsibility |  |  |
| I believe we have a responsibility to choose sustainable transportation to protect the environment | 0.96 |  |  |
| I believe we have a moral responsibility to choose sustainable transportation | 0.95 |  |  |
| I don’t think the responsibility to choose sustainable transportation is on me | 0.65 |  |  |
| Thinking about how individual values influence the decision to choose sustainable transportation, how much do you agree with the following statements: |  |  |  |
| I want to use sustainable transportation because I care for the environment | 0.87 |  |  |
| My values do not have an impact on my decision to choose sustainable transportation | 0.58 |  |  |
| Alpha (α) | 0.90 |  |  |

*Cross-loading item omitted.

**Loading below 0.3.

***The correlations are presented in the order the factors appear (i.e., factor 1 with 2, factor 1 with 3 and so on)

****Discarded as KMO value was less than .5 and analysis rerun.

*****Discarded to increase alpha reliabilities from 0.78 to 0.86.

******Discarded to increase alpha reliabilities from 0.69 to 0.81.

Factor analyses of beliefs underlying use of public transportation

| **Belief** | **Factor 1** | **Factor 2** | **Factor 3** | **Factor 4** |
| --- | --- | --- | --- | --- |
| **Behavioural beliefs** |  |  |  |  |
| Choosing public transportation on a regular basis would… | Disadvantages | Environmental benefits | Positive experience | Practical advantages |
| …help the environment |  | 0.91 |  |  |
| …reduce my carbon footprint |  | 0.88 |  |  |
| …lead to less air pollution |  | 0.66 |  |  |
| …lower my transportation costs |  |  |  | 1.04 |
| …be convenient |  |  |  | 0.36 |
| …lead to traffic reduction |  |  | 0.45 |  |
| …be enjoyable |  |  | 0.98 |  |
| …be easy* |  |  |  |  |
| …be relaxing |  |  | 0.70 |  |
| …be expensive* |  |  |  |  |
| …be time consuming | 0.80 |  |  |  |
| …be unpleasant | 0.49 |  |  |  |
| …be stressful | 0.66 |  |  |  |
| …be inconvenient | 0.88 |  |  |  |
| …reduce my freedom and flexibility | 0.89 |  |  |  |
| …be unreliable. | 0.76 |  |  |  |
| Factor correlations*** | -0.44, 0.06, 0.54 | -0.44, -0.32,-0.61 | 0.06, -0.32, 0.08 | 0.54, -0.61, 0.08 |
| Alpha (α) | 0.87 | 0.85 | 0.77 | 0.72 |
|  |  |  |  |  |
| **Normative beliefs** |  |  |  |  |
| The following individuals or groups think I should choose public transportation on a regular basis: | Approvers | Positive role models | Negative role models |  |
| Environmentalists |  | 0.54 |  |  |
| Government** |  |  |  |  |
| General public | 0.65 |  |  |  |
| Commuters | 0.94 |  |  |  |
| Young people | 0.57 |  |  |  |
| People with easy public transport access | 0.53 |  |  |  |
| City workers | 0.60 |  |  |  |
| The following individuals or groups think I should NOT choose public transportation on a regular basis: |  |  |  |  |
| People who live in rural areas** |  |  |  |  |
| The following people or groups of people are most likely to use public transportation on a regular basis: |  |  |  |  |
| People on lower incomes |  | 0.64 |  |  |
| Students |  | 0.85 |  |  |
| Elderly |  | 0.44 |  |  |
| School pupils |  | 0.60 |  |  |
| Commuters | 0.38 |  |  |  |
| People without cars |  | 0.53 |  |  |
| The following people or groups of people are least likely to use public transportation on a regular basis: |  |  |  |  |
| Wealthy |  |  | 0.77 |  |
| Drivers |  |  | 0.88 |  |
| Rural residents |  |  | 0.58 |  |
| Factor correlations*** | -0.18, -0.39 | -0.18, -0.25 | -0.39, -0.25 |  |
| Alpha (α) | 0.79 | 0.75 | 0.77 |  |
|  |  |  |  |  |
| **Control beliefs** |  |  |  |  |
| It would be easier for me to choose public transportation if public transportation was… | Facilitators | Ineffectiveness | Inconvenience |  |
| …more available | 0.69 |  |  |  |
| ….more reliable | 0.70 |  |  |  |
| …cheaper | 0.80 |  |  |  |
| …more convenient | 0.83 |  |  |  |
| …more comfortable | 0.73 |  |  |  |
| …more frequent | 0.83 |  |  |  |
| The following factors or circumstances would make it harder for me to choose public transportation: |  |  |  |  |
| High price | -0.51 |  |  |  |
| Low accessibility |  | 0.84 |  |  |
| Low availability |  | 0.91 |  |  |
| Unreliability |  | 0.68 |  |  |
| Inconvenience |  |  | 0.76 |  |
| Time consuming |  |  | 0.87 |  |
| Travelling a long distance |  |  | 0.53 |  |
| Factor correlations*** | -0.28, 0.30 | -0.28, -0.07 | 0.30, -0.07 |  |
| Alpha (α) | 0.88 | 0.89 | 0.72 |  |
|  |  |  |  |  |
| **Habit beliefs** |  |  |  |  |
| Thinking about your behaviour over the last few years, how much do you agree with the following statements: | Experience | Automatic choice |  |  |
| I tried public transportation but it took too long | 0.63 |  |  |  |
| I tried public transportation and it was easy and fast | 0.44 |  |  |  |
| I tried public transportation but it was uncomfortable | 0.53 |  |  |  |
| I tried public transportation but it was inconvenient | 0.78 |  |  |  |
| I tried public transportation but it was inconvenient | 0.71 |  |  |  |
| I have not tried public transportation |  | 0.40 |  |  |
| I found myself automatically choosing public transportation when... |  |  |  |  |
| …I went on a day or weekend trip |  | 0.55 |  |  |
| …I travelled long distances |  | 0.48 |  |  |
| …it was convenient |  | 0.94 |  |  |
| …I travelled in city centres |  | 0.58 |  |  |
| Factor correlations | -0.18 | -0.18 |  |  |
| Alpha (α) | 0.76 | 0.70 |  |  |
|  |  |  |  |  |
| **Moral beliefs** |  |  |  |  |
| Thinking about individual responsibility to use public transport, how much do you agree with the following statements: | Moral responsibility |  |  |  |
| I believe I have a responsibility to use public transport as it is better for the environment | 0.83 |  |  |  |
| I don’t think I have a responsibility to use public transport as I want to maximise my comfort | 0.71 |  |  |  |
| I don’t think the responsibility to choose sustainable transportation is on me | 0.64 |  |  |  |
| Thinking about how individual values may influence the decision to use public transportation, how much do you agree with the following statements: |  |  |  |  |
| My values don’t influence my decision to use public transport** |  |  |  |  |
| I want to use public transportation because I care for the environment | 0.67 |  |  |  |
| Alpha (α) | 0.80 |  |  |  |

*Cross-loading item omitted.

**Loading below 0.3.

***The correlations are presented in the order the factors appear (i.e., factor 1 with 2, factor 1 with 3 and so on)

Factor analyses of beliefs underlying walking and cycling

| **Belief** | **Factor 1** | **Factor 2** | **Factor 3** | **Factor 4** |
| --- | --- | --- | --- | --- |
| **Behavioural beliefs** |  |  |  |  |
| Walking and/or cycling on a regular basis would... | Advantages | Disadvantages |  |  |
| ...give me a chance to exercise | 0.57 |  |  |  |
| ...improve my health | 0.65 |  |  |  |
| ...help me save money** |  |  |  |  |
| ...make me feel connected to the nature | 0.59 |  |  |  |
| ...be good for the environment | 0.49 |  |  |  |
| ...reduce carbon emissions | 0.55 |  |  |  |
| ...give me a sense of accomplishment | 0.75 |  |  |  |
| ...make me happy | 0.77 |  |  |  |
| ...make me feel healthy | 0.79 |  |  |  |
| ...improve my wellbeing | 0.83 |  |  |  |
| ...make me feel relaxed | 0.62 |  |  |  |
| ...be time consuming |  | 0.52 |  |  |
| ...be tiring |  | 0.64 |  |  |
| ...be affected by bad weather |  | 0.58 |  |  |
| ...can make my health deteriorate* |  |  |  |  |
| ...be inconvenient |  | 0.82 |  |  |
| ...be dangerous |  | 0.50 |  |  |
| ...be frustrating |  | 0.78 |  |  |
| Factor correlations*** | -0.09 | -0.09 |  |  |
| Alpha (α) | 0.88 | 0.82 |  |  |
|  |  |  |  |  |
| **Normative beliefs** |  |  |  |  |
| The following individuals or groups think I should walk and/or cycle on a regular basis: | Positive role models | Approvers | Disapprovers | Negative role models |
| Medical professionals | 0.86 |  |  |  |
| Environmentalists | 0.78 |  |  |  |
| Politicians |  | 0.31 |  |  |
| Fit people | 0.69 |  |  |  |
| Everyone |  | 0.72 |  |  |
| My family |  | 0.70 |  |  |
| Teenagers |  | 1.03 |  |  |
| Young people |  | 0.94 |  |  |
| The following people or groups of people think I should NOT walk and/or cycle on a regular basis: |  |  |  |  |
| Elderly |  |  | 0.95 |  |
| People with health issues |  |  | 1.00 |  |
| People with disabilities |  |  | 0.82 |  |
| The following people or groups of people are most likely to walk and/or cycle on a regular basis: |  |  |  |  |
| Athletes | 0.37 |  |  |  |
| Healthy people | 0.42 |  |  |  |
| People concerned about the environment | 0.39 |  |  |  |
| Young people | 0.31 |  |  |  |
| Children** |  |  |  |  |
| People who live close to school/work | 0.59 |  |  |  |
| Ramblers** |  |  |  |  |
| The following people or groups of people are least likely to walk and/or cycle on a regular basis: |  |  |  |  |
| Lazy people | -0.37 |  |  |  |
| People with long distances to travel | -0.35 |  |  |  |
| Parents |  |  | 0.38 |  |
| Elderly |  |  |  | 0.71 |
| People with disabilities |  |  |  | 0.97 |
| People with limited mobility |  |  |  | 0.82 |
| People who do not have enough time* |  |  |  |  |
| Workers** |  |  |  |  |
| Factor correlations*** | 0.06, 0.17, -0.28 | 0.06, 0.12, -0.39 | 0.17, 0.12, 0.30 | -0.28, -0.39, 0.30 |
| Alpha (α) | 0.79 | 0.87 | 0.85 | 0.82 |
|  |  |  |  |  |
| **Control beliefs** |  |  |  |  |
| The following factors or circumstances would make it easier for me to walk and/or cycle on a regular basis: | Facilitators | Personal barriers | External barriers |  |
| Short distance to travel | 0.72 |  |  |  |
| Better infrastructure (e.g., cycle lanes, walking paths) | 0.88 |  |  |  |
| Cheaper bicycles** |  |  |  |  |
| City/town characteristics (e.g., flat terrain) | 0.73 |  |  |  |
| Good weather | 0.46 |  |  |  |
| Safety | 0.47 |  |  |  |
| Being physically fit | 0.64 |  |  |  |
| The following factors or circumstances would make it harder for me to walk and/or cycle on a regular basis: |  |  |  |  |
| Traffic** |  |  |  |  |
| Disability |  | 0.91 |  |  |
| Health issues |  | 1.00 |  |  |
| Bad weather |  |  | 0.98 |  |
| Distance |  |  | 0.67 |  |
| City/town characteristics |  |  | 0.48 |  |
| Factor correlations*** | 0.22, 0.47 | 0.22, 0.53 | 0.47, 0.53 |  |
| Alpha (α) | 0.81 | 0.95 | 0.78 |  |
|  |  |  |  |  |
| **Habit beliefs** |  |  |  |  |
| Thinking about your behaviour over the last few years, how much do you agree with the following statements: | Automatic choice | Positive experience | Negative experience |  |
| I tried walking and/or cycling but it took too long |  |  | 0.52 |  |
| I tried walking and/or cycling but I could not cope due to my health |  |  | 0.92 |  |
| I tried walking and/or cycling and it had a positive impact on my health and wellbeing |  | 1.05 |  |  |
| I tried walking and/or cycling and I enjoyed it |  | 0.54 |  |  |
| I found myself automatically choosing sustainable transportation when... |  |  |  |  |
| I go shopping | 0.58 |  |  |  |
| Distance is manageable | 0.92 |  |  |  |
| I travel locally | 0.85 |  |  |  |
| Factor correlations*** | 0.38, -0.40 | 0.38, -0.16 | -0.40, -0.16 |  |
| Alpha (α) | 0.80 | 0.75 | 0.62 |  |
|  |  |  |  |  |
| **Moral beliefs** |  |  |  |  |
| Thinking about individual responsibility to walk and/or cycle, how much do you agree with the following statements: | Responsibility |  |  |  |
| I believe walking and/or cycling is a personal preference** |  |  |  |  |
| I believe we have a responsibility to walk and/or cycle as they are better for the environment | 0.76 |  |  |  |
| Thinking about how individual values affect the decision to walk and/or cycle, how much do you agree with the following statements: |  |  |  |  |
| I want to walk and/or cycle because I value my health and body | 0.60 |  |  |  |
| My values do not have an impact on my decision to choose walking and/or cycling** |  |  |  |  |
| I want to walk and/or cycle because I care for the environment | 0.75 |  |  |  |
| Alpha (α) | 0.74 |  |  |  |

*Cross-loading item omitted.

**Loading below 0.3.

***The correlations are presented in the order the factors appear (i.e., factor 1 with 2, factor 1 with 3 and so on)

Factor analyses of beliefs underlying car use reduction

| **Belief** | **Factor 1** | **Factor 2** | **Factor 3** | **Factor 4** |
| --- | --- | --- | --- | --- |
| **Behavioural beliefs** |  |  |  |  |
| Reducing my car use on a regular basis would... | Disadvantages | Environmental benefits | Negative feelings | Health benefits |
| …help the environment |  | 0.89 |  |  |
| …reduce my carbon footprint |  | 0.83 |  |  |
| …lead to less air pollution |  | 0.91 |  |  |
| …lower my transportation costs* |  |  |  |  |
| …make me choose healthier alternatives |  |  |  | 1.01 |
| …make me feel healthy |  |  |  | 0.60 |
| …be unpleasant |  |  | 1.02 |  |
| …be inconvenient |  |  | 0.73 |  |
| …require more planning | 0.65 |  |  |  |
| …lead to a reduction of freedom | 0.72 |  |  |  |
| …make me feel isolated | 0.45 |  |  |  |
| …make me annoyed |  |  | 0.61 |  |
| …require more time spent travelling | 0.60 |  |  |  |
| …limit the distance I can travel | 1.06 |  |  |  |
| Factor correlations*** | 0.35, 0.18, 0.39 | 0.35, 0.09, 0.35 | 0.18, 0.09, 0.68 | 0.39, 0.35, 0.68 |
| Alpha (α) | 0.80 | 0.92 | 0.81 | 0.81 |
|  |  |  |  |  |
| **Normative beliefs** |  |  |  |  |
| The following individuals or groups think I should reduce my car use on a regular basis: | Positive role models | Negative role models |  |  |
| Environmentalists | 0.34 |  |  |  |
| Young people** |  |  |  |  |
| General public** |  |  |  |  |
| The following people or groups of people think I should NOT reduce my car use on a regular basis: |  |  |  |  |
| Emergency services** |  |  |  |  |
| Petrol companies** |  |  |  |  |
| Parents** |  |  |  |  |
| The following people or groups of people are most likely to reduce their car use on a regular basis: |  |  |  |  |
| People who live in a city centre** |  |  |  |  |
| Climate activists | 0.86 |  |  |  |
| People concerned about the environment | 0.96 |  |  |  |
| Elderly** |  |  |  |  |
| People on lower incomes** |  |  |  |  |
| The following people or groups of people are least likely to reduce their car use on a regular basis: |  |  |  |  |
| Elderly** |  |  |  |  |
| Rural workers |  | 1.00 |  |  |
| Wealthy people |  | 0.56 |  |  |
| Factor correlations*** | 0.16 | 0.16 |  |  |
| Alpha (α) | 0.74 | 0.73 |  |  |
|  |  |  |  |  |
| **Control beliefs** |  |  |  |  |
| The following factors or circumstances would make it easier for me to reduce my car use on a regular basis: | Barriers - poor alternatives | Facilitators | Barriers - practical difficulties |  |
| Better public transport |  | 0.68 |  |  |
| Reduced price of public transport |  | 1.04 |  |  |
| The following factors or circumstances would make it harder for me to reduce my car use on a regular basis: |  |  |  |  |
| Lack of regular public transport | 0.97 |  |  |  |
| Lack of reliable public transport | 0.93 |  |  |  |
| Work being too far |  |  | 0.33 |  |
| Price |  |  | 0.96 |  |
| Factor correlations*** | 0.08, -0.15 | 0.08, -0.38 | -0.15, -0.38 |  |
| Alpha (α) | 0.92 | 0.82 | 0.52 |  |
|  |  |  |  |  |
| **Habit beliefs** |  |  |  |  |
| Thinking about your behaviour over the last few years, how much do you agree with the following statements: | Experience | Negative experience |  |  |
| I tried reducing my car use and I had a positive experience | 0.89 |  |  |  |
| I tried reducing my car use and it was hard and unreliable |  | 0.88 |  |  |
| I tried reducing my car use and it saved money | 0.72 |  |  |  |
| I tried reducing my car use but it was not good due to wasting too much time |  | 0.90 |  |  |
| I have not tried reducing my car use due to inconvenience | 0.58 |  |  |  |
| I found myself automatically reducing my car use when I went... |  |  |  |  |
| …on school runs | 0.52 |  |  |  |
| …on local trip | 0.54 |  |  |  |
| …on long journeys | 0.54 |  |  |  |
| …food shopping | 0.53 |  |  |  |
| Factor correlations*** | 0.14 | 0.14 |  |  |
| Alpha (α) | 0.80 | 0.87 |  |  |
|  |  |  |  |  |
| **Moral beliefs** |  |  |  |  |
| Thinking about individual responsibility to reduce car use, how much do you agree with the following statements: | Moral Responsibility |  |  |  |
| I believe I have a responsibility to reduce my car use to protect the environment | 0.86 |  |  |  |
| I do not think I have a responsibility to reduce my car use because I need a car | 0.71 |  |  |  |
| I do not think I have a responsibility to reduce my car use because other people don't | 0.80 |  |  |  |
| Thinking about how individual values may influence your decision to reduce car use, how much do you agree with the following statements: |  |  |  |  |
| I want to reduce my car use because I care for the environment. | 0.86 |  |  |  |
| My values do not have an impact on my decision to reduce my car use. | 0.61 |  |  |  |
| Alpha (α) | 0.87 |  |  |  |

*Cross-loading item omitted.

**Loading below 0.3.

***The correlations are presented in the order the factors appear (i.e., factor 1 with 2, factor 1 with 3 and so on)

Factor analyses of beliefs underlying flights reduction

| **Belief** | **Factor 1** | **Factor 2** | **Factor 3** | **Factor 4** |
| --- | --- | --- | --- | --- |
| **Behavioural beliefs** |  |  |  |  |
| Reducing my flights on a regular basis would… | Negative personal impact | Environmental benefits | Inconvenience |  |
| ...help the environment |  | 0.97 |  |  |
| ...reduce my carbon footprint |  | 0.80 |  |  |
| ...lead to less air pollution |  | 0.91 |  |  |
| ...lower my transportation costs** |  |  |  |  |
| ...make me feel eco-friendly |  | 0.65 |  |  |
| ...make me feel financially secure** |  |  |  |  |
| ...make me happy* |  |  |  |  |
| ...not have any advantages* |  |  |  |  |
| ...require longer travelling time |  |  | 0.66 |  |
| ...be inconvenient |  |  | 1.11 |  |
| ...affect my social and family network | 0.61 |  |  |  |
| ...lead to less or no travel abroad | 0.59 |  |  |  |
| ...have a negative impact on work** |  |  |  |  |
| ...make me feel like I am missing out | 0.91 |  |  |  |
| ...limit my freedom | 0.83 |  |  |  |
| ...affect my mental health | 0.90 |  |  |  |
| Factor correlations*** | 0.34, -0.67 | 0.34, -0.29 | -0.67, -0.29 |  |
| Alpha (α) | 0.84 | 0.88 | 0.76 |  |
|  |  |  |  |  |
| **Normative beliefs** |  |  |  |  |
| The following individuals or groups think I should reduce my flights on a regular basis: | Negative role models | Approvers | Eco-approvers | Positive role models |
| People who are concerned about the environment |  |  | 0.91 |  |
| Climate activists |  |  | 1.00 |  |
| Business people |  | 0.95 |  |  |
| Employers |  | 0.90 |  |  |
| Everybody |  | 0.72 |  |  |
| The following people or groups of people think I should NOT reduce my flights on a regular basis: |  |  |  |  |
| Airlines* |  |  |  |  |
| Solo travellers** |  |  |  |  |
| My family** |  |  |  |  |
| The following people or groups of people are most likely to reduce their flights on a regular basis: |  |  |  |  |
| Businesses |  | 0.45 |  |  |
| Climate activists |  |  |  | 0.75 |
| People concerned about the environment |  |  |  | 1.01 |
| People on lower incomes** |  |  |  |  |
| The following people or groups of people are least likely to reduce their flights on a regular basis: |  |  |  |  |
| Business people | 0.67 |  |  |  |
| Famous people | 0.97 |  |  |  |
| Wealthy people | 1.00 |  |  |  |
| People who deny climate change | 0.81 |  |  |  |
| Factor correlations*** | 0.08, -0.18, 0.03 | 0.08, -0.36, 0.04 | -0.18, -0.36, 0.26 | 0.03, 0.04, 0.26 |
| Alpha (α) | 0.91 | 0.85 | 0.96 | 0.85 |
|  |  |  |  |  |
| **Control beliefs** |  |  |  |  |
| The following factors or circumstances would make it easier for me to reduce my flights on a regular basis: | Barriers - Worse alternatives | Facilitators - Better alternatives | Work |  |
| Cheaper alternatives |  | 1.02 |  |  |
| Efficient alternatives |  | 0.80 |  |  |
| Going on local holidays** |  |  |  |  |
| Work circumstances changing |  |  | 1.02 |  |
| The following factors or circumstances would make it harder for me to reduce my flights on a regular basis: |  |  |  |  |
| Lack of alternatives | 1.00 |  |  |  |
| Worse alternatives in terms of cost and practicality | 0.82 |  |  |  |
| Work requirements |  |  | -0.40 |  |
| Family pressure** |  |  |  |  |
| Factor correlations*** | -0.17, -0.36 | -0.17, 0.27 | -0.36, 0.27 |  |
| Alpha (α) | 0.90 | 0.89 | 0.61 |  |
|  |  |  |  |  |
| **Habit beliefs** |  |  |  |  |
| Thinking about your behaviour over the last few years, how much do you agree with the following statements: | Experience |  |  |  |
| I tried reducing my flights and it was good | 0.93 |  |  |  |
| I have not tried to reduce my flights as I enjoy travelling | 0.70 |  |  |  |
| I found myself automatically choosing to reduce my flights in the following circumstances: |  |  |  |  |
| Work situations | 0.48 |  |  |  |
| When it is expensive | 0.33 |  |  |  |
| None | 0.43 |  |  |  |
| Alpha (α) | 0.72 |  |  |  |
|  |  |  |  |  |
| **Moral beliefs** |  |  |  |  |
| Thinking of individual responsibility to reduce flights, how much do you agree with the following statements: | Responsibility |  |  |  |
| I believe I have a responsibility to reduce flights to protect the environment | 0.80 |  |  |  |
| The decision to reduce flights is an individual choice** |  |  |  |  |
| I don't believe reducing flights is a responsibility as I don't fly often enough | 0.54 |  |  |  |
| Thinking of how individual values influence the decision to reduce flights, how much do you agree with the following statements: |  |  |  |  |
| I want to reduce flights because I care for the environment | 0.80 |  |  |  |
| My values do not have an impact on my decision to reduce flights | 0.47 |  |  |  |
| I want to reduce flights because I am conscientious | 0.71 |  |  |  |
| I don't want to reduce my flights because I put myself and my wellbeing first | 0.55 |  |  |  |
| Alpha (α) | 0.81 |  |  |  |

*Cross-loading item omitted.

**Loading below 0.3.

***The correlations are presented in the order the factors appear (i.e., factor 1 with 2, factor 1 with 3 and so on)
